# Suspicious Minds: Unexpected Election Outcomes, Perceived Electoral Integrity and Satisfaction With Democracy in American Presidential Elections

**DOI:** 10.1177/10659129231166679

**Published:** 2023-04-10

**Authors:** Philippe Mongrain

**Affiliations:** 1 McGill University, Montreal, QC, Canada

**Keywords:** elections, expectations, integrity, satisfaction with democracy, United States, winner–loser gap

## Abstract

A great amount of research has noted the existence of a gap between election winners and losers in relation to perceptions of electoral fairness and satisfaction with democracy. One aspect of the winner–loser gap that has been overlooked is the impact of citizens’ expectations about election outcomes on these attitudes. More precisely, how do citizens react to unexpected defeats and victories? Are individuals on the losing side less critical of the electoral process or dissatisfied with democracy when they recognize beforehand that their favourite party or candidate was likely to be defeated? Does experiencing a surprise victory lead to a boost in perceived electoral integrity or democratic satisfaction? To answer these questions, I use data from the 1996, 2000, 2004, 2012, 2016 and 2020 ANES. While there is little evidence that expectations exert a major influence on post-election attitudes, outcome unexpectedness seems to have decreased confidence in the vote counting process among losers, independents and even winners in the 2020 election. The results show the considerable influence that fraud claims and conspiracy theories can have on public opinion when elected officials and candidates push a consistent story line of electoral malfeasance and corruption in an effort to denigrate political opponents.

## Introduction

Regularly held elections are one of the main mechanisms to regulate conflicts in democratic societies (Przeworski 2018). Provided that the rules are fair, it is expected that citizens will accept the outcome of an election whether they are on the winning side or the losing side (Anderson et al. 2005). Hence, citizens’ confidence in the electoral process is an essential condition for the stability and longevity of democratic institutions as well as the peaceful alternation of power.

The violent protests that preceded and followed the 2020 American presidential election, based on the claim that the electoral process was fraudulent and that victory had been ‘stolen’ from President Trump, are a powerful reminder of democracy’s reliance on citizens’ willingness to accept the outcome of an election as legitimate. Both in 2016 and 2020, Donald Trump entertained a dual set of expectations among his supporters: he would win easily and the other side would resort to all sorts of ‘dirty tricks’ on an unprecedented scale. Throughout his presidency, Trump has supported a number of voter-fraud conspiracy theories, including rigged voting machines, counterfeited and dumped mail-in ballots, stuffed ballot boxes in Democrat-controlled cities and votes cast in the name of deceased people. The narrative of election theft in the 2020 presidential campaign has encouraged ‘Stop the Steal’ protests and rallies that culminated in the United States Capitol attack of January 6, 2021, during which a crowd of pro-Trump supporters disrupted the joint session of Congress assembled to count electoral votes by storming the Capitol building. Justwan and Williamson (2022) found that exposure to election fraud claims was convincing enough to reduce Trump voters’ faith in electoral fairness. As Eggers, Garro and Grimmer (2021, 6) noted, ‘[t]he Trump campaign delivered a blueprint for losing candidates to undermine support for the winner or even steal the election. It seems unlikely that he will be the last to try these tactics’.

In light of previous academic work, the reaction of Trump supporters following his failed reelection bid is hardly surprising, although unparalleled in magnitude. A great amount of research has noted the existence of a gap between winners and losers in relation to perceptions of electoral fairness, satisfaction with democracy (SWD), trust in political institutions and other attitudes. As stated by Craig et al. (2006, 579), ‘winners and losers do not always respond with equal enthusiasm either to the election outcome, or to the institutions and processes through which that outcome was rendered’. Those who are part of the majority (i.e. citizens who voted for the governing party or one of the parties in government) are generally more inclined to say that they are satisfied with the way democracy works than those in the minority (i.e. citizens who supported a losing party) (Anderson et al. 2005; Henderson 2008; Sinclair, Smith and Tucker 2018; Singh, Karakoç and Blais 2012). Anderson et al.’s (2005) results also showed less support for democratic principles and a higher willingness or potential to engage in political protest among losers.

One important aspect of the winner–loser gap that has been overlooked is the impact of citizens’ expectations about election outcomes and their perceptions of electoral integrity. More precisely, how do citizens react to unexpected victories and losses? Are individuals on the losing side less critical of the electoral process or dissatisfied with democracy when they recognize that their favourite party or candidate was likely to be defeated at the polls? Does experiencing a surprise victory lead to a boost in perceived electoral fairness or democratic satisfaction? Leiter, Reilly and Stegmaier (2020, 6) have claimed that ‘when faced with a reality that does not comport to their established beliefs, citizens may react quite negatively—an increasingly concerning phenomenon in modern democracies’. However, this claim has yet to be fully tested in the context of electoral competition. Understanding which factors influence the size and nature of the winner–loser gap is of paramount importance since losers’ consent is a crucial condition for the stability of democratic institutions. The existence of a gap suggests that elections do not fully succeed in conferring legitimacy upon the institutions of representative democracy. According to Nadeau and Blais (1993, 553), ‘the viability of electoral democracy depends on its ability to secure the support of a substantial proportion of individuals who are displeased with the outcome of an election’. In other words, sore losers, if they are numerous enough, might pose a real threat to democracy. This can be of particular concern since the effects of winning or losing are not necessarily short-lived and might consolidate through repeated experiences (Anderson et al. 2005; Chang, Chu and Wu 2014; Dahlberg and Linde 2016; 2017; Daniller and Mutz 2019). The proliferation of misinformation and false news (or allegations of fake news) could also create unrealistic expectations among certain segments of the general public. As mentioned by Loveless (2021a, 66), ‘fake news offers a means for some to circumvent a reality which disallows others’ preferred outcome’.

There are strong reasons to believe that disconfirmed expectancies, at least among losers, produce negative emotions: by losing when expecting to win, one has to put up with both the unpleasantness of unpredictability and an outcome that does not match his or her own preference (Mandler 1975). Therefore, the disappointment from an unexpected loss has a potentially more detrimental impact on perceptions of integrity and satisfaction with democracy than it does when one correctly senses that his or her preferred party is on its way to defeat. In a similar fashion, winning when one expects to lose should enhance individuals’ feelings of electoral fairness and satisfaction with democracy. Previous research has repeatedly found that electoral expectations were in great part driven by partisan biases (e.g. Delavande and Manski 2012; Dolan and Holbrook 2001). As a consequence, whether an election outcome is unexpected should also partly result from these same biases. Many voters will anticipate the victory of their preferred party or candidate even in the face of unfavourable odds or when exposed to discrepant polling information (Kuru, Pasek and Traugott 2020; Madson and Hillygus 2020).^1^ In a context of growing partisan polarization across a number of advanced democracies, including the United States (Boxell, Gentzkow and Shapiro 2021; Dalton 2021; Layman, Carsey and Horowitz 2006), and mounting concerns about political disinformation (Sindermann, Cooper and Montag 2020; Tandoc 2019), unexpected outcomes could imply increasingly large winner–loser gaps in political attitudes. However, the potential association between (unfulfilled) expectations and attitudes toward the electoral process and the functioning of democracy has been given very little attention.

The goal of this paper is to explore the influence of voters’ expectations on their perceptions of electoral integrity and their level of satisfaction with democracy in the context of U.S. presidential elections using American National Election Study data collected between 1996 and 2020. Generally speaking, the results are encouraging for democracy. In most of the elections covered by the present study, unexpectedness does not seem to profoundly shape individuals’ post-election attitudes. However, the existence of a gap in perceived electoral integrity among all categories of respondents (losers, independents and winners) due to unexpectedness in the aftermath of the 2020 election emphasizes just how the sustained ‘rigged election’ discourse of that campaign might have negatively impacted public perceptions of fairness across various segments of the population and raises serious concerns for future elections.

## Disconfirmed Expectancies and Political Attitudes

Numerous studies show that perceptions of integrity as well as satisfaction with democracy are heavily influenced by the outcome of elections (see, e.g. Levy 2021; Sances and Stewart 2015). According to Anderson et al. (2005, 39), ‘losers are more likely to take a critical view of a process that did not favor them’. Therefore, beliefs in election meddling are in great part explained by motivated partisan reasoning. Losers are more prone to report wrongdoing because they feel cheated (Edelson et al. 2017; Grant et al. 2021; Sances and Stewart 2015; Sinclair, Smith and Tucker 2018; Vail et al. 2022). Partisan biases and ‘double-standards’ can be particularly strong as Beaulieu (2014, 31) concluded that American voters ‘tend not to be concerned about election integrity if their party benefits from potential fraud’. Moreover, current research on affective polarization shows that supporters and candidates of opposing parties are increasingly seen as unworthy of trust (Iyengar et al. 2019), which does nothing to alleviate suspicions of fraudulent behaviour from out-group partisans. Beliefs in election fraud have also been found to correlate with conspiratorial thinking, populist attitudes and anti-social personality traits (Edelson et al. 2017; Enders et al. 2021; Norris, Garnett and Grömping 2020; Sinclair, Smith and Tucker 2018). According to Edelson et al. (2017, 939), ‘conspiratorial predispositions strongly predict the belief that if one’s candidate were to lose, fraud would have been involved’.

Furthermore, as already mentioned, losers tend to express less satisfaction with the functioning of the political system than winners. According to Chang, Chu and Wu (2014, 235), ‘[t]his proposition is built upon the simple intuition that the political system is more favourable to people whose supported party is in power, and that individuals tend to favour rules that make them win, whereas losers may attribute their defeat to the rules of the electoral game’. In the case of American presidential elections, one could make the argument that an unexpected loss in the 2000 and 2016 elections, in which the popular vote winner was defeated, is more likely (or as likely) to impact respondents’ satisfaction with democracy than it is to influence their perceptions of electoral integrity. Voters, particularly those on the losing side, might consider it unfair for a candidate to win more votes nationwide than his or her main opponent and still lose the election. In other political contexts, important discrepancies between seat and vote shares have been found to decrease support for existing voting rules among supporters of both minor and major parties (Plescia, Blais and Högström 2020). Since Al Gore’s loss in 2000, Democrats have been much more supportive of amending the Constitution (i.e. change the way democracy works) so the candidate who receives the most total votes nationwide wins the election (Brenan 2020; Salzer and Kiley 2022). It is therefore important to consider both the impact of unexpectedness on perceived electoral integrity and satisfaction with democracy.

However, existing research has hardly considered the potential role of voters’ expectations, and particularly unmet expectations, in explaining their sentiments toward democracy and electoral fairness. In other words, are unexpected losers’ and winners’ reactions and attitudes different from those of other voters? The literature in psychology suggests that people who show optimism in relation to a particular outcome are more likely to exhibit higher disappointment than pessimistic individuals when reality does not live up to their expectations (Sweeny and Shepperd 2010). In general, the higher the optimism, the higher the cost of disconfirmed expectancies. In other words, the strength of individuals’ expectations should mitigate their reaction: greater certainty in a particular outcome should lead to greater disappointment in the face of disconfirmation. Perhaps, Miceli and Castelfranchi (2015, 43–46 – italics in original) best summarize the psychological effects of unfulfilled expectations:

The disconfirmation of a positive expectation looks like a *violation*, and elicits a feeling of mistreatment, as if one were suffering some injustice. The stronger the positive expectation, the stronger this sense of unfairness. [. . . ] When a negative expectation is invalidated, the expectant will of course be surprised, but is very unlikely to protest or feel mistreated. Actually, one will feel relieved and happy, because one’s own goal *p* has been fulfilled.

The ‘expectancy-disconfirmation’ model (EDM) formalizes these theoretical expectations. As mentioned above, it has often been observed that wrongly anticipating an outcome tends to create a state of psychological discomfort, which might even breed denial. It is most surprising that this well-known fact has remained almost ignored in studies of voters’ post-election attitudes. The EDM framework is a well-known paradigm in research on consumer satisfaction in the private sector (Anderson 1973; Cardozo 1965) and has increasingly been applied to public services (Zhang et al. 2022). The main idea behind this model is simple: ‘[i]nitial expectations frame a customer’s mind […], so that if performance is significantly worse than the comparison standard, a customer will experience negative disconfirmation. On the contrary, if perceived performance exceeds expectation, positive disconfirmation results’ (Au and Tse 2019 292). There is also the possibility of ‘zero disconfirmation’ when actual performance matches the customer’s expectations (Oliver 2010, 100–101). For example, Tse (2003) has shown that positive discrepancies in food and service quality were linked to higher tips, while negative discrepancies led to lower tips. Interestingly, unexpected bad catering experiences were ‘punished’ more heavily than unexpected good experiences were rewarded. In fact, the greater effect of negative disconfirmation compared to positive disconfirmation is consistent with findings of a negativity bias in consumer evaluations (Darke, Ashworth and Main 2010; Li, Meng and Pan 2020). Generally speaking, negative information or outcomes weigh more heavily in people’s judgements and decisions than corresponding positive information or outcomes (Soroka, Fournier and Nir 2019). Negativity bias has been described by Baumeister et al. (2001, 362) as ‘one of the most basic and far-reaching psychological principles’.

**Figure 1.**
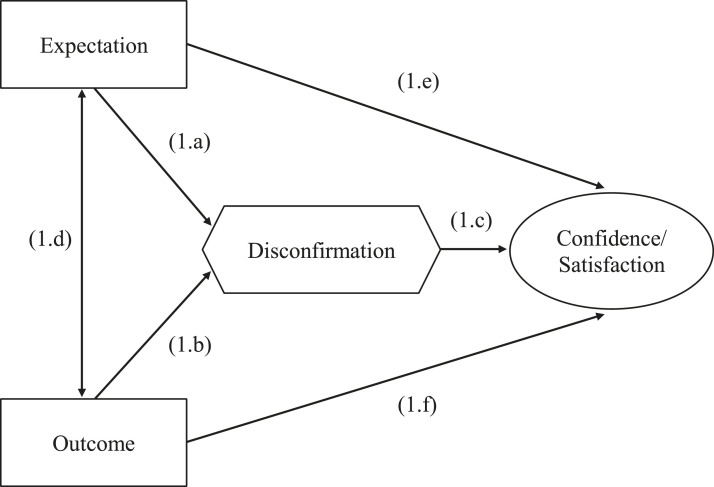
Expectancy-Disconfirmation Model. *Note.* Figure inspired by Van Ryzin (2004).

Figure 1 provides a visual representation of the expectancy-disconfirmation model with perceived electoral integrity and satisfaction with democracy as the judgements of interest. This being a model of customer satisfaction at its roots, some conceptual clarifications are in order. As mentioned earlier, in marketing research, disconfirmation corresponds to the gap between the expected and perceived performance of a product or service. In the case of an election, the ‘product’ is the candidate or party that is supported by a citizen. Perceptions about the performance of a candidate or party can be a matter of degree or it can involve a simple dichotomous evaluation. As shown in Figure 1, disconfirmation is jointly influenced by one’s expectation or forecast (link 1.a) and by the outcome (link 1.b) – since disconfirmation results from the gap between these two elements. Higher expectations are more likely to produce negative disconfirmation. The nature of the disconfirmation will affect one’s level of confidence in the electoral process (link 1.c). An outcome that exceeds one’s expectation or matches their expectation^2^ will lead to greater confidence or satisfaction, while a disappointing outcome will result in lower confidence or satisfaction. Links 1.a, 1.b and 1.c represent the core expectancy-disconfirmation model (Oliver 2010). It is also possible that expectations and perceptions of performance are correlated: having high expectations could lead an individual to judge an outcome more positively (link 1.d). However, the direction of this potential relationship is not specified. Finally, both expectations and the perceived outcome can exert a direct influence on confidence (links 1.e and 1.f, respectively). In the present paper, the interest lies mainly in the core model (i.e. links 1.a, 1.b and 1.c) since its constituent elements revolve entirely around the gap between expectations and the observed outcome.

Miceli and Castelfranchi (2015) make an important distinction between one’s pseudo-goal of seeing a prediction confirmed and one’s internal goal of seeing a preferred outcome realized. When an individual’s negative expectation (for example, forecasting the loss of one’s favourite party) turns out to be wrong, the disconfirmed expectancy could generate cognitive distress (because there is a gap between expectations and reality); however, since the internal goal (i.e. the victory of one’s favourite party) has been achieved, it would only be natural for the individual who has been proven wrong to feel relief and joy. Nevertheless, it is probable that unexpected positive outcomes have less of an effect than unexpected negative outcomes due to individuals’ tendency to reflect more heavily on negative events than positive ones as mentioned above.

Only a few scholars have directly addressed the potential association between citizens’ expectations and post-election attitudes. Blais and Gélineau’s (2007) analysis of voters’ satisfaction with democracy in the 1997 Canadian federal election did not support the idea that ‘optimistic’ (i.e. unexpected) losers would be more dissatisfied than ‘pessimistic’ (i.e. expected) losers. Neither did they find winners expecting a loss to be more satisfied with democracy after the election than winners expecting a victory. Blais and Gélineau (2007) nonetheless noted that this was ‘a surprising finding since there are plausible theoretical reasons to suppose that one’s reaction to the outcome of an election hinges on one’s prior expectations’. Using data from the 2012 American National Election Study, Hollander (2014) provided mixed evidence regarding the influence of unexpected losses on individuals’ attitudes toward politics and the political system: while surprised losers were no more dissatisfied with democracy or trusting of government than expected losers, they were more likely to perceive the government as a threat and to express lower levels of confidence in the electoral process. Umit (2020) showed that satisfaction with democracy was lower among ‘Remain’ supporters in the 2016 Brexit referendum who believed in victory compared to Remainers expecting to lose. This was particularly true among losers with high victory expectations. Interestingly, Schaffner (2020) only found gaps in *pre-evaluations* of electoral fairness between both Remainers and Leavers according to their expectations. However, ‘[a]fter the referendum, outcome expectations no longer [had] a significant moderating effect on the perceived fairness of the referendum’ (Schaffner 2020, 293).

Note that two of the four papers that have directly looked at the association between expectations and system support attitudes only considered differences between winners and losers in terms of satisfaction with democracy. As Linde and Ekman (2003) point out (and as acknowledged by Blais and Gélineau 2007), the SWD question should not be interpreted as an indicator of citizens’ beliefs about the legitimacy of the democratic system or their acceptance of its guiding principles. The SWD item is mostly a measure of the perceived performance of the democratic regime. Whereas SWD is perhaps closer to an indicator of system performance, perceived electoral fairness might be a clearer measure of citizens’ confidence (or lack thereof) in the process deciding who gets to hold power, one of the core elements (if not the core element) of representative democracy (Flesken and Hartl 2020). Norris, Frank and Martínez i Coma (2015, 6) state that ample empirical evidence exists to support ‘the claim that perceptions of the honesty or fairness of elections are indeed commonly linked with social-psychological indicators of legitimacy, including low or declining feelings of trust in elected authorities, lack of confidence in the fairness of electoral procedures and authorities, and skepticism about the honesty of the outcome’. Citizens’ evaluations of electoral integrity are not without consequences: for example, individuals who perceive elections to be fair are more likely to vote than those who see flaws in the electoral process (Birch 2010; Sinclair, Smith and Tucker 2018). Norris (2014) has also demonstrated that perceptions of electoral malpractices tend to encourage protest activism such as attending peaceful demonstrations or joining political strikes. Perceived problems with elections might also adversely affect democratic satisfaction (Norris 2019). The 2020 U.S. presidential election has also shown that these same perceptions of unfairness and injustice could lead to outright violence (Kydd 2021; Piazza 2022; Pope 2022).

Despite mixed findings, existing research provides enough theoretical arguments to posit three related hypotheses describing the potential links between expectations and perceptions of electoral integrity as well as satisfaction with democracy, that is:

Hypothesis 1 Unexpected losers will display less confidence in the electoral process and satisfaction with democracy than losers who correctly predicted the defeat of their favourite candidate.

Hypothesis 2 Unexpected winners will display greater confidence in the electoral process and satisfaction with democracy than winners who correctly predicted the victory of their favourite candidate.

Hypothesis 3 As the gap between expectations and reality increases, so too will the positive (negative) effect of unfulfilled expectation on confidence in the electoral process and satisfaction with democracy for unexpected winners (losers).

In light of the strong evidence pointing toward an asymmetry between positive and negative information, a fourth hypothesis can be stated:

Hypothesis 4 The effect of unexpected defeats will be greater than that of unexpected victories (in other words, we should find more support for *H1* than *H2*).

## Data and Methods

Data were drawn from the 2020 American National Election Study (ANES). The 2020 ANES was specifically chosen because it includes both a post-election measure of satisfaction with democracy and a pre- and post-election measure of perceived electoral integrity alongside the necessary information regarding voters’ expectations. With a pre-/post-election panel design, it is possible to control for prior levels of perceived electoral integrity and, hence, isolate more precisely the impact of respondents’ expectations (Blais and Gélineau 2007, 428). The ability to measure pre-levels of perceived electoral integrity means that we can assess change in respondents’ attitudes before and after the election. This brings us closer to a causal demonstration (Hillygus and Snell 2018). Due to the unusual amount of fraud claims made by President Trump and his supporters, the 2020 election is probably one of the most interesting cases available to study citizens’ perceptions of electoral fairness. At the same time, it begs the question of just how generalizable the insights and lessons from this election can be. In order to better contextualize the results obtained from the 2020 ANES, I also leverage additional data from the 1996, 2000, 2004, 2012 and 2016 presidential elections (see section A of the online appendix for exact data sources; the Supplemental appendix can be found under ‘Supplementary Material’ on *PRQ*’s website). All models were estimated via ordinary least squares regressions. Table 1 below presents an overview of U.S. presidential election outcomes and ANES respondents’ forecasts between 1996 and 2020.^3^

**Table 1.** Overview of U.S. Presidential Election Outcomes and Citizens’ Forecasts, 1996–2020*.

Election	Outcome	% 2-Party Vote	% Electoral College	% Correct Forecasts
US 1996	Bill Clinton (D)	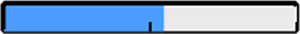	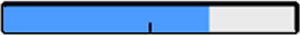	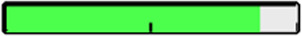
US 2000	George W. Bush (R)	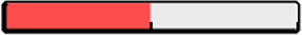	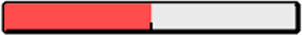	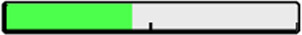
US 2004	George W. Bush (R)	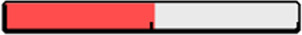	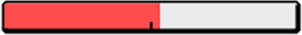	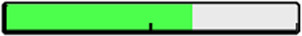
US 2012	Barack Obama (D)	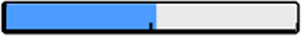	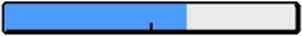	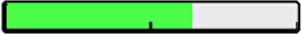
US 2016	Donald Trump (R)	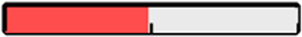	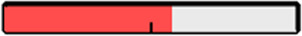	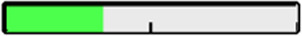
US 2020	Joe Biden (D)	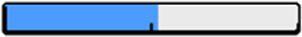	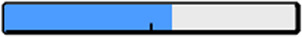	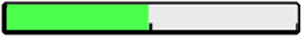

*Note*. Election results retrieved from The American Presidency Project. Percentage of correct citizens’ forecasts computed using American National Election Study data. Blue denotes the outcome for Democratic candidates (D) and red denotes the outcome for Republican candidates (R). *2008 election excluded.

Note that post-election interviews between 1996 and 2020 were generally conducted from the day following the election until the end of December/January.^4^ The replication materials for this article are deposited in the Harvard Dataverse at https://doi.org/10.7910/DVN/HLEIAH.

### Dependent Variables

As mentioned above, the present paper investigates the impact of unmet expectations on (a) perceived electoral integrity and (b) satisfaction with democracy. Perceptions of electoral fairness were measured differently across surveys. Respondents of the 1996–2004 ANES were presented with the following statement and question: ‘In some countries, people believe their elections are conducted fairly. In other countries, people believe that their elections are conducted unfairly. Thinking of the last election in the United States, where would you place it on this scale of one to five where 1 means that the last election was conducted fairly and 5 means that the last election was conducted unfairly’? In the 2012–2020 ANES, respondents were asked the following question: ‘In your view, how often do the following things occur in this country’s elections: Votes are counted fairly’? There were four possible answer categories in 2012 (i.e. very often, fairly often, not often or not at all often) and five in 2016 and 2020 (i.e. all of the time, most of the time, about half of the time, some of the time or never). The five-point scale was reduced to a four-point scale by merging two categories (i.e. ‘about half of the time’ and ‘some of the time’). These questions were asked in the post-election surveys. Since the perceived electoral integrity item in the 2012–2020 ANES refers to *all* elections conducted in the U.S. rather than the most recent general election, one might think that a specific election was rigged or unfair without necessarily believing that all elections conducted in the near or distant past were flawed. This means that we run the risk of *underestimating* the association between unexpectedness and perceptions of electoral integrity. It is also possible that citizens’ overall evaluation of electoral integrity in the U.S. is mostly influenced by their views on the most recent election. This would be in line with Zaller’s (1992, 49 – italics added) response axiom, according to which ‘individuals answer survey questions by averaging across the considerations that are *immediately salient or accessible* to them’.

Satisfaction with democracy was measured in all six ANES post-election surveys by the standard question: ‘On the whole, are you very satisfied, fairly satisfied, not very satisfied, or not at all satisfied with the way democracy works in the United States’? The dependent variables were coded so that the highest value represents the highest level of perceived electoral integrity or satisfaction with democracy.

### Independent Variables

Voters’ expectations about the outcome of the presidential election constitute the main independent variable of interest. ANES respondents were simply asked who they believed would be elected president in the November election (i.e. the Democratic candidate, the Republican candidate or another candidate). To assess the impact of expectations on perceptions of electoral integrity and satisfaction with democracy, an interaction term between the electoral status of respondents (i.e. losers, independents/others, winners) and a binary variable indicating whether the result was expected (i.e. correct forecasts) or unexpected (i.e. incorrect forecasts and ‘don’t know’ answers) was created. Respondents’ (pre-election wave) party identification was used in order to determine whether an individual was on the losing side or the winning side (leaners were considered as partisans). As a robustness check, all the analyses were reran using respondents’ reported vote instead of party identification to distinguish winners from losers. These additional analyses are available in the Supplemental appendix. No major differences in the findings were noted.

The strength of voters’ expectations was measured through one item asking respondent whether they believed the presidential race would be close or the candidate they predicted as winner would ‘win by quite a bit’ (predictions of noncompetitive races were coded 1 and predictions of close elections were coded 0). Unexpected losses should lead to greater suspicion about electoral integrity when losers expected an easy victory for their preferred candidate. In contrast, unexpected winners might experience a boost in perceived electoral integrity if they believed the opposing candidate would win easily.

Being a winner or a loser (or expecting to be so) is, obviously, only one of the many factors that can influence one’s perceptions of electoral integrity or satisfaction with democracy. As mentioned earlier, variables related to ‘suspiciousness’ often positively correlate with beliefs in electoral fraud and wrongdoing. Since there is no credible evidence of massive election fraud in American elections (Eggers, Garro and Grimmer 2021; Minnite 2011), any belief stating otherwise can be described as conspiratorial in nature. Conspiratorial thinking is mainly about the lack of trust in established authorities and ‘official’ explanations of events. Conspiracy theories imply that small groups of powerful individuals act in secret, hiding the truth from the general public for their own political or economic benefit (Douglas et al. 2019; Uscinski, Klofstad and Atkinson 2016). The 2012, 2016 and 2020 ANES each have a small battery of items related to conspiratorial thinking or ‘non-mainstream’ beliefs. While the 2020 ANES includes broader indicators of conspiratorial thinking (e.g. ‘Much of what people hear in schools and the media are lies designed to keep people from learning the real truth about those in power’), belief in conspiracy theories in the 2012 and 2016 ANES was assessed in relation to specific events or topics (e.g. Barack Obama’s birthplace). In each case, these items were used to create a conspiratorial thinking index (see also section E of the Supplemental appendix). Unfortunately, no such measures were available in the 1996–2004 ANES.

Individuals who hold cynical views of politics should also, logically, show less confidence in the electoral process and be less satisfied with democracy than others (Karp, Nai and Norris 2018). In the present paper, political cynicism is an index composed of different items. Only items included in the pre-election surveys were considered as unfulfilled expectations could have an influence on post-electoral levels of political cynicism (but see section I of the Supplemental appendix). An additional measure of suspicious mindsets was added to the models, namely, social mistrust (i.e. the general lack of trust in other individuals). Once again, such pre-election measures were only found in the 2012–2020 ANES.^5^ The specific items used are presented in section B of the Supplemental appendix. Apart from the variables presented above, I also control for respondents’ age, gender, race, level of education, income and factual political knowledge. Economically and socially advantaged groups (i.e. those who are highly educated, have higher incomes and men) are potentially more satisfied with the way democracy and institutions work. Karp, Nai and Norris (2018) found that politically sophisticated voters were more likely to trust the electoral process. People with high education are also less likely to believe in conspiracy theories (van Prooijen 2017). Furthermore, these control variables are especially important as unexpectedness itself might be influenced by sociodemographic characteristics (although the evidence is mixed) as well as political sophistication (Mongrain 2021; Morisi and Leeper 2022). All models also include region fixed effects. Since economic evaluations could also shape citizens’ opinions about regime performance (Campbell 2015; Christmann 2018; Quaranta and Martini 2016), SWD models include respondents’ retrospective and prospective egotropic and sociotropic economic evaluations.

Finally, in the 2020 ANES, perceptions of electoral integrity were measured both before and after the election. A pre-post model was created for the 2020 presidential election. In the pre-election survey, respondents were asked how accurately (i.e. not at all accurately, a little accurately, moderately accurately, very accurately or completely accurately) they thought votes would be counted in the general election. Although the pre- and post-election measures of electoral integrity are not exactly the same, they are similar enough to ensure that a statistically significant interaction between respondents’ electoral status and their expectations is not due to the inability to control for pre-existing attitudes about electoral fairness.

## Results

### Unexpected-Expected Gap

**Figure 2.**
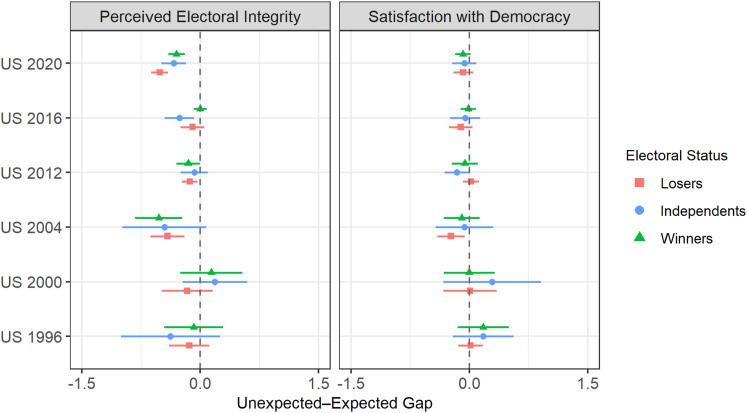
Unexpected-Expected Gap in Perceived Electoral Integrity and Satisfaction with Democracy According to Respondent’s Electoral Status, 1996–2020. *Note.* The vertical lines represent the 95% confidence intervals.

Figure 2 displays the main results.^6^ The left-side panel shows the size of the unexpected-expected gap in perceived electoral integrity between losers, independents and winners for each election. This gap corresponds to the average difference between individuals who incorrectly predicted the outcome and those who correctly anticipated the outcome among each group of respondents (i.e. losers, independents/others and winners).

In the first set of elections (1996–2004; fairness of last election), perceptions of electoral integrity only appear to matter in the 2004 presidential election. In 1996, the vast majority of ANES respondents correctly predicted Bill Clinton’s reelection. During the entire campaign, Clinton maintained a significant lead in vote intention polls over his Republican opponent. In light of the low variation in outcome ‘unexpectedness’ in 1996, the absence of statistically significant results is not surprising.

The statistically insignificant results for the 2000 election could be explained by the delayed announcement of the outcome (Halliez and Thornton 2022). Although there is little doubt that the recount dispute in Florida and Al Gore’s defeat, despite being the popular vote winner, were important sources of discontent among Democratic voters, the 2000 ANES post-election survey (which asked respondents to rate the fairness of the electoral process) was in the field for a period of several weeks during which no president-elect had been named, which might have been a cause of confusion or uncertainty among voters. We should also consider the possibility that some winners (i.e. Republican supporters) were irritated by the legal challenges to George W. Bush’s victory. The results for the 2004 election are somewhat surprising. The defeat of John Kerry in 2004 fuelled conspiracy theories and allegations of widespread voting irregularities, most notably election improprieties by the Ohio Republicans that could have cost Kerry the presidency (Gumbel 2008, 1112). The statistically significant and negative gap in perceived electoral integrity between unexpected and expected losers (−0.42 points) is conform to *H1*. However, the statistically significant and negative gap in perceived electoral integrity between unexpected and expected winners (−0.53 points) is contrary to *H2* and harder to explain. Republicans who believed George W. Bush might be a one-term president were apparently less trustful than their more ‘optimistic’ fellow partisans. In the second set of elections (2012–2020; fair vote count), we find additional evidence in support of *H1*. In the 2012 and 2020 presidential elections, unexpected losers were less confident in the electoral process than expected losers (−0.13 points and −0.51 points, respectively). Interestingly, as in 2004, unexpected winners in these two elections were apparently more suspicious of the electoral process than expected winners (−0.15 points and −0.30 points, respectively). The size of the gap between unexpected and expected losers (but also independents and winners) is particularly impressive in 2020 as respondents were asked how frequently were votes counted fairly in American elections rather than solely in the most recent presidential election.^7^ The results for the 2016 election are puzzling for at least three reasons. Firstly, and as can be seen in Table 1, only a third of the ANES sample (less than 4.5% of Democrats, 4.3% of independents and 25% of Republicans) correctly predicted Donald Trump’s victory, testifying to the surprising nature of this election both among losers *and* winners, which, in theory, should have elicited strong reactions. Vote intention polls and projections by poll aggregators, with odds largely in favour of Clinton, probably contributed to the surprise (Kennedy et al. 2018). Secondly, in addition to Trump’s bombastic campaign style and outlandish statements, the 2016 election was particularly contentious with both sides raising serious concerns about the fairness of the campaign: claims of so-called ‘zombie voters’ and illegal immigrants casting ballots by millions, assertions of biased media coverage and fears of Russian interference were all dominant features of the 2016 campaign. Finally, it was the second time in less than 20 years that Democratic supporters had to accept the defeat of their candidate despite the poorer popular vote performance of the Republican nominee, which could have impacted perceptions of electoral fairness.

The right-side panel of Figure 2 displays the results for satisfaction with democracy. As can be seen, there is practically no evidence of a difference between voters according to their expectations. Generally speaking, in terms of perceptions of electoral integrity, I find only limited support in favour of *H1* and absolutely no support in favour of *H2*: where significant gaps were identified, unexpected winners were *more*, not less, suspicious about electoral integrity than expected winners. Note also that the results do not really provide any supporting evidence for *H4*: in general, the size of the gap between unexpected and expected winners does not seem substantially smaller than that between unexpected and expected losers, notwithstanding the fact that unexpected winners do not appear to react positively to the disconfirmation of their expectations.

### Expected Election Closeness

As already mentioned, the strength of citizens’ post-election attitudes could also depend on how certain they were about their expectations. ANES respondents were asked whether they believed the outcome of the presidential election would be close or not. Citizens expecting an easy victory or even a landslide for their favourite candidate should be the most disappointed when facing defeat and the most inclined to question the legitimacy of the outcome. Dividing losers, independents and winners according to the strength of their expectations provides little evidence in support of *H3*. Only for the 1996 and 2020 elections do we find statistically significant differences. In 2020, the gap between unexpected and expected losers is greater among those who wrongly perceived the election as a relatively noncompetitive one for their favourite candidate (−0.88 points vs −0.35 points).

Gaps between unexpected and expected losers that are not apparent in Figure 2 also reveal themselves once perceptions of election closeness are accounted for. In 1996, Republican partisans who believed Bob Dole would easily defeat President Clinton were considerably more likely to express lower levels of perceived electoral fairness than their copartisans who predicted an easy victory for Clinton (−1.33 points). There is no such gap, however, among Republicans who predicted a hard-fought victory for either Clinton or Dole. Finally, in 2020, Republicans who believed Trump would be easily reelected expressed, on average, considerably less satisfaction with democracy than their copartisans who predicted an easy victory for Biden (−0.49 points). Once again, there is no such gap among Republicans who predicted a hard-fought victory for either Biden or Trump. See section F of the Supplemental appendix for related figures.

### Pre-Post Design

All of the previous analyses rely on a post-election measure of perceived electoral integrity and satisfaction with democracy. The 2020 ANES affords us the rare opportunity of using a pre-post design on beliefs about electoral integrity. Unfortunately, such is not the case for satisfaction with democracy. In the 2020 ANES pre-election survey, respondents were asked how accurately they thought votes would be counted in the November general election. Table C.1.7 in the Supplemental appendix displays the coefficients from two models. Both of these models include the pre-election measure of integrity. The first model includes none of the controls used previously (i.e. conspiracy beliefs, political cynicism, social mistrust, political knowledge, etc.), while the second model integrates these controls. The gaps between unexpected and expected losers, independents and winners found earlier remain statistically and substantively significant in the two models displayed in Table C.1.7. Voters in the 2020 American presidential election who did not anticipate defeat (i.e. mostly Republican supporters) did lower, on average, their evaluation of electoral integrity after election day in comparison to voters who expected to lose by approximately 0.52 points. Decreases in perceptions of electoral integrity were less pronounced among independents and winners who failed to predict Biden’s victory, but they were non-negligible (−0.34 points and −0.28 points, respectively). This is as one would expect considering how widespread allegations of electoral malpractices and systematic voter fraud coming from President Trump and his allies were during and after the campaign (Benkler et al. 2020). Trump consistently claimed that he would win in a landslide, while at the same time pushing the idea that a Biden victory would clearly indicate electoral wrongdoing from the Democratic Party. Several polls conducted in the months following the 2020 presidential election have found that a majority of Republicans believed Joe Biden’s victory was not legitimate (Cuthbert and Theodoridis 2022).

## Discussion and Conclusion

Concerns over growing partisan resentment and hostility are being voiced regularly. Although a great share of research on political polarization focuses specifically on the American case, divisive partisan politics has become a widespread phenomenon with daunting consequences for multiple established and new democracies. As Carothers and O’Donohue (2019, 2) point out, ‘[i]t exacerbates intolerance and discrimination, diminishes societal trust, and increases violence throughout the society’. Therefore, how citizens handle displeasing and unexpected political outcomes should be a prime area of research. Unmet electoral expectations have the potential to create deficits in perceived legitimacy. In a classic study, Festinger, Riecken and Schachter (1956) investigated how cult members responded to failed prophecies. They observed that some members of a small apocalyptic UFO religion in Chicago (the ‘Seekers’) became stronger believers when their prediction of a catastrophic event was disconfirmed – disciples were supposed to be saved by extraterrestrial aliens from a great flood that would engulf much of the U.S. territory.

When reality does not live up to our expectations, we normally experience emotions such as disappointment, regret, frustration and even more serious forms of mental distress (see, e.g. Culatta and Clay-Warner 2021; Polivy and Herman 2000; Seyd 2016; Zeelenberg, van Dijk, Manstead and van der Pligt 2000). With growing polarization in the United States (Pierson and Schickler 2020), it seems that backlash-type reactions in the face of dissonant information, such as the one described by Festinger, Riecken and Schachter (1956), are becoming a common occurrence in American politics. As an example, Madson and Hillygus (2020) found that voters were becoming more extreme on their prior opinions when presented with disconfirming polling results. Another recent study by Harmon-Jones, Harmon-Jones and Denson (2020) showed that the dissonance caused by exposure to the alleged wrongdoings of one candidate (i.e. Donald Trump) incited supporters to emphasize the wrongdoings of his opponent (i.e. Hillary Clinton).

In the context of an election, surprise losses should have a detrimental influence on citizens’ attitudes toward politics. Unexpected election victories should have the opposite effect. Pierce, Rogers and Snyder (2016, 45) have already noted that ‘[e]lection outcomes strongly affect the short-term happiness/sadness of partisan losers, with minimal impact on partisan winners’. Although the impact of election losses on happiness can be somewhat short-lived, some studies have shown that decreases in satisfaction with democracy and trust can persist for the entire duration of a government’s term among losers and can gradually generate entrenched dissatisfaction through repeated experiences (Anderson et al. 2005; Chang, Chu and Wu 2014; Loveless 2021b; see also Schaffner 2020).

That being said, the results of the present study are reassuring for democracy: in most cases, disconfirmed expectancies do not seem to exert a determining influence on voters’ confidence in the electoral process or on their overall level of satisfaction with the system. Most importantly, surprised losers do not appear more likely than other voters to question the legitimacy of election outcomes. Results from the 2020 American presidential elections are, however, especially worrisome. These results suggest that politicians and elected officials can successfully create (or aggravate) a confidence crisis among voters by pushing a consistent narrative of fraud and wrongdoing from political opponents. During the 2020 campaign, Donald Trump nurtured two contrasting sets of expectations, namely, that (1) he would win ‘big’ and that (2) Democrats would do all they could to steal the election. It is no wonder, then, that Trump supporters who believed in his victory were particularly upset with the electoral process after Joe Biden’s victory. Persuading citizens that the system is rigged or unfair (whether or not this is actually the case), could strengthen the adverse effects of losing on political trust (Mauk 2022). The use of voter suppression and voter-fraud allegations as mobilization tactics by Democrats and Republicans is a well-known phenomenon and there seems to be an important ‘disconnection between the amount of voter fraud in American elections and the rhetoric surrounding the issue’ both among the general public and in terms of media coverage (Fogarty et al. 2015, 5). These tactics are quite clearly something of a double-edged sword and may severely harm democracy for the sake of short-sighted partisan gains (Albertson and Guiler 2020). And, worryingly, fact-checking does not seem to be of much help in countering the effect of dubious allegations (Barrera, Guriev, Henry and Zhuravskaya 2020; Berlinski et al. 2021). In fact, Graham and Svolik (2020) have argued that the commitment to democratic principles among the American public is generally weak and highly bendable, which affords an extremely limited protection against illiberal tendencies. According to them, ‘voters are willing to trade off democratic principles for partisan ends’ (Graham and Svolik 2020, 407). Worryingly, allegations and actual revelations of electoral fraud are met with a great deal of partiality. One study among Danish and Mexican voters showed that, although partisans strongly disapproved foul play from their own party (as much as they did for other parties), revelations of electoral malpractice were usually not enough to cause voters to defect from their party (Aarslew 2022). In other words, strong partisans were more supportive of an in-party government that engaged in election meddling than they were of an out-party government that won fair and square.

Although the present study relies on data from multiple elections, it is limited to a single country, one where two parties largely dominate the political sphere and where affective and ideological polarization has been increasing in the last election cycles. I believe the results provide valuable insights for research on citizens’ expectations and their perceptions of electoral integrity and satisfaction with democracy, but they do not allow us to make inferences on the potential role of institutional factors in moderating voters’ beliefs about election outcomes and their consequences on disconfirmed expectancies.

## Supplemental Material

Supplement material - Suspicious Minds: Unexpected Election Outcomes, Perceived Electoral Integrity and Satisfaction With Democracy in American Presidential Elections Click here for additional data file. Supplemental material for Suspicious Minds: Unexpected Election Outcomes, Perceived Electoral Integrity and Satisfaction With Democracy in American Presidential Elections by Philippe Mongrain in Political Research Quarterly

## Notes

1. The role played by polls in the formation of voters’ expectations is addressed in section H of the Supplemental appendix.

2. It is unclear whether confirmed low expectations should lead to actual satisfaction or more neutral sentiments.

3. The 2008 ANES is excluded because it does not have any measure of perceived electoral integrity.

4. For more details on the timeline and distribution of post-election interviews, see Figure A.1 in the Supplemental appendix.

5. Social mistrust was measured in the pre-election wave of the 1996 ANES, but was not included in the main model for 1996 to keep all analyses for the 1996–2004 elections as similar as possible.

6. Detailed regression tables for each election are available in the Supplemental appendix (see section C). Section D of the Supplemental appendix presents additional analyses that include a three-way interaction between electoral status, unexpectedness, and an index made up of all suspicious thinking items.

7. The statistically significant gap in perceived electoral integrity between unexpected and expected losers in 2004 and 2020 is found among both strong and weak/leaner partisans. The negative gap found among unexpected and expected winners in 2004 and 2020 remains statistically significant among weak partisans and leaners, but is either insignificant or barely significant among strong partisans (see section G of the Supplemental appendix).
