# Supplementary material for: Suspicious Minds: Unexpected Election Outcomes, Perceived Electoral Integrity and Satisfaction With Democracy in American Presidential Elections
Source: Polit Res Q. 2023 Apr 10;76(4):1589–603. doi: 10.1177/10659129231166679 (PMC10615620; doi:10.1177/10659129231166679)
Supplement: Supplement material - Suspicious Minds: Unexpected Election Outcomes, Perceived Electoral Integrity and Satisfaction With Democracy in American Presidential Elections [file sj-pdf-1-prq-10.1177_10659129231166679.pdf]

Appendix

Suspicious Minds: Unexpected Election Outcomes, Perceived Electoral Integrity, and Satisfaction with Democracy in American Presidential Elections

Philippe Mongrain

Contents

A Data **1**

B Variables **3**
- B.1 Dependent Variables . . . 3
- B.2 Expectations . . . 3
- B.3 Electoral Status . . . 4
- B.4 Suspicion . . . 5
- B.5 Economic Evaluations . . . 8
- B.6 Political Knowledge . . . 9
- B.7 Sociodemographics . . . 10
- B.8 Descriptive Statistics . . . 13

C Regression Tables: 2-Way Interaction **16**
- C.1 Party Identification . . . 16
- C.2 Reported Vote . . . 22

D Regression Tables: 3-Way Interaction **28**
- D.1 Party Identification . . . 29
- D.2 Reported Vote . . . 33

E Conservative vs Liberal Conspiracy Beliefs **37**
- E.1 Party Identification . . . 38
- E.2 Reported Vote . . . 40

F Expected Closeness **42**
- F.1 Party Identification . . . 42
- F.2 Reported Vote . . . 43

G Party Identification Strength **44**

H The Influence of Polls **45**

I Suspicious Thinking: Additional Details and Analyses **49**
- I.1 Party Identification . . . 49
- I.2 Reported Vote . . . 55

References **61**

A Data

Results for the 1996, 2000, 2004, 2012, 2016, and 2020 U.S. presidential elections can be found here: The American Presidency Project. Election studies data were gathered from the American National Election Study website:

- 1996 Time Series Study. See American National Election Study (1996).
- 2000 Time Series Study. See American National Election Study (2000).
- 2004 Time Series Study. See American National Election Study (2004).
- 2012 Time Series Study. See American National Election Study (2012).
- 2016 Time Series Study. See American National Election Study (2016).
- 2020 Time Series Study. See American National Election Study (2020).

Figure A.1 on the following page shows the daily number of post-election interviews for each election. Although the outcome of the 2016 U.S. presidential election stirred a great deal of controversy fuelled by claims of "deceased people" and non-citizens voting, possible foreign interference in the campaign as well as a potential "Comey effect" (i.e., the potentially detrimental impact of FBI Director James Comey's October 28 letter to the House Judiciary Committee regarding Clinton's emails on her campaign), Hillary Clinton conceded defeat to Donald Trump the day after the election once the Republican candidate had reached the required 270 electoral votes to become president. In 2000 and 2020, concession speeches by the losing candidates came much later as the certainty and legitimacy of the outcome were questioned and subjected to legal scrutiny. In 2020, the Trump campaign preemptively launched bids to challenge state results although the outcome of the election was not known until November 7. Over the days and months following the election, dozens of lawsuits claiming election fraud and irregularities were filed by the Trump administration, with almost no success, in state and federal courts. A detailed timeline of events for the 2020 election and previous presidential contests is beyond the scope of the present paper, but at least two elements can be noted: (1) in 2000, the vast majority of respondents were questioned before the *Bush v. Gore* decision[1]; and (2) in 2020, all respondents were questioned before the January 6, 2021 Capitol attack and Trump's concession speech the following day. Furthermore, no interviews were conducted before the major networks called Pennsylvania for Biden, giving him the needed majority of electoral votes to win the election. Therefore, the result of the 2020 election was known when the ANES post-election interviews began (although Trump supporters might have believed in his ability to upend the election up until the beginning of January 2021). This was not the case in 2000: the outcome of the election was not known for more than a month until the Supreme Court ruled that the recount of presidential ballots in Florida be stopped. This could be an issue as Halliez and Thornton (2022, 7) observed that "[g]enuine uncertainty about results delays judgments about democracy." In other words, the winner-loser gap in satisfaction with democracy in the aftermath of the 2000 election was only apparent among those interviewed after Gore's concession (see also Craig et al. 2006). Unfortunately, the 2000 ANES data do not really offer any satisfying solution in that regard.

[1] Al Gore conceded defeat to George W. Bush on December 13, 2000 after the *Bush v. Gore* case was decided. Therefore, it would seem appropriate to keep only those respondents who were interviewed after Gore's concession. Unfortunately, only a small sample of ANES respondents (too small for meaningful analyses) was interviewed after this date.

Figure A.1. Daily Distribution of Post-Election Interviews, 1996–2020 ANES

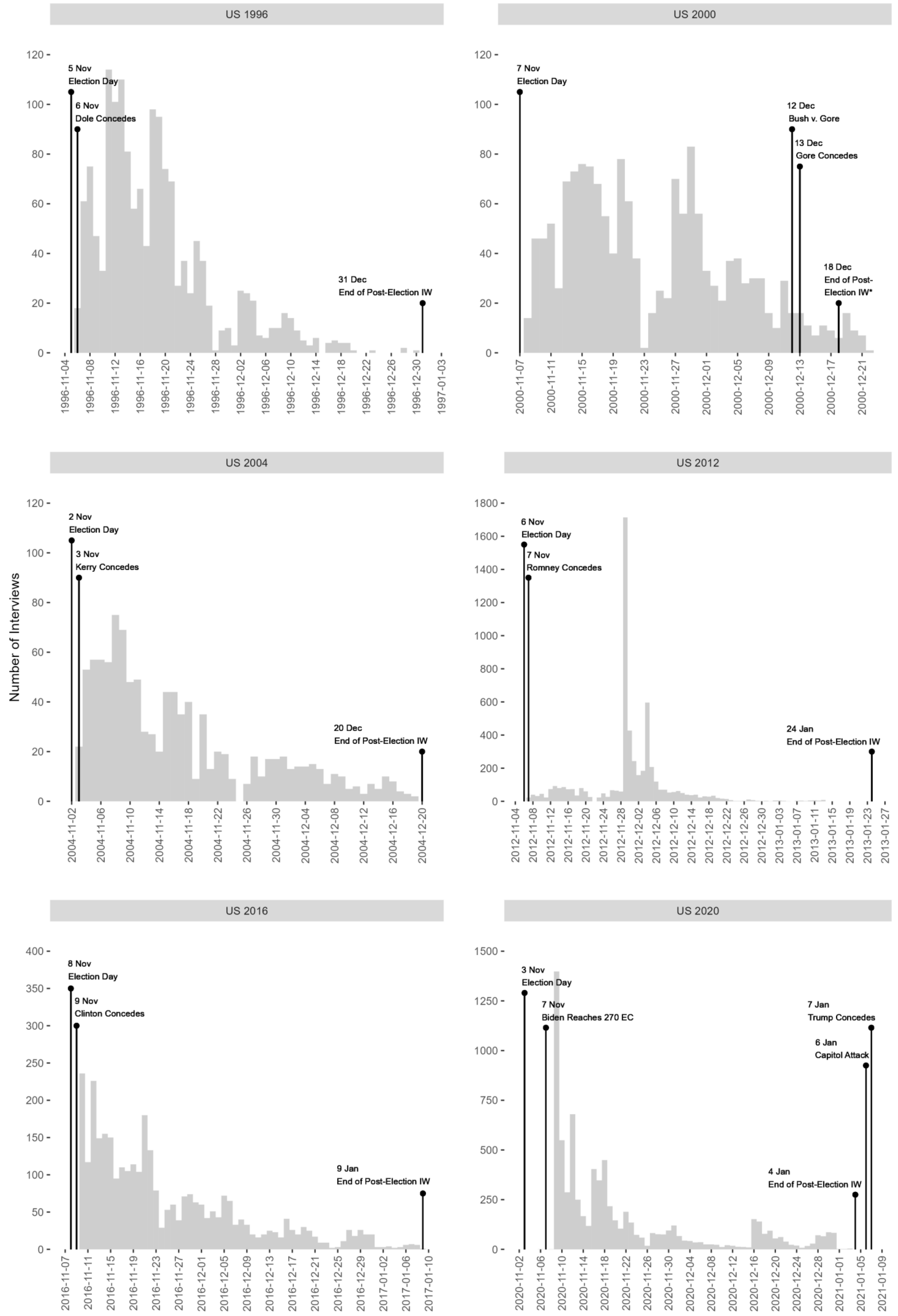

Note. For a detailed chronology of events in the aftermath of the 2000 U.S. presidential election, see U.S. Election Atlas or Stanford Law Library. *Although the ANES website mentions December 18 as the last day of post-election interviewing in 2000, it seems as if a small number of respondents were interviewed between December 19 and 22.

B Variables

B.1 Dependent Variables

Perceived electoral integrity. Perceived electoral integrity was measured on a four-point scale. The five-point scale used in the 2012 and 2016 ANES was reduced to a four-point scale by merging two categories (i.e., "about half of the time" and "some of the time").

– *1996–2004 U.S. presidential elections.* In some countries, people believe their elections are conducted fairly. In other countries, people believe that their elections are conducted unfairly. Thinking of the last election in the United States, where would you place it on this scale of one to five where 1 means that the last election was conducted fairly and 5 means that the last election was conducted unfairly?

– *2012 U.S. presidential election.* In your view, how often do the following things occur in this country's elections: Votes are counted fairly? Does this occur very often, fairly often, not often, or not at all often?

– *2016–2020 U.S. presidential elections.* In your view, how often do the following things occur in this country's elections: Votes are counted fairly? All of the time, most of the time, about half of the time, some of the time, or never?

Satisfaction with democracy. Satisfaction with democracy was measured on a four-point scale.

– *1996–2020 U.S. presidential elections.* On the whole, are you (very) satisfied, fairly satisfied, not very satisfied, or not at all satisfied with the way democracy works in the United States?

B.2 Expectations

Expected winner. Respondents' electoral expectations were coded 0 for incorrect forecasts (unexpected outcomes), including "don't know" answers, and 1 for correct forecasts (expected outcomes). The present study is, obviously, not without its limitations. A possible limitation of the present paper is the inability to distinguish optimism/pessimism from expectations as there might be a disconnect between one's hopes and what they think is most likely to happen (e.g., expecting your favourite candidate to lose, but believing s/he has a shot). This could have important consequences for the post-election attitudes of losers (but see section I of the appendix). In their study of unexpected election outcomes and satisfaction with democracy in the 1997 Canadian federal election, Blais and Gélineau (2007) use the terms "optimistic winners/losers" and "pessimistic winners/losers" to classify voters: "[v]oters were construed to be 'optimistic' when the chances of winning they gave their party in either the national or constituency contests were at least 50 per cent, and to be pessimistic otherwise" (Blais and Gélineau, 2007, 433). It is unclear if Blais and Gélineau (2007) truly make a conceptual distinction between optimism and expectations, but their definition cannot be applied to citizens' forecasts from the ANES as these forecasts were in a purely qualitative format (Who will be elected?) rather than a probabilistic one (How likely is it that...?), notwithstanding the fact that a 50 per cent chance of winning in a multiparty or two-party-plus system (like Canada) probably does not have the same meaning as it does in an almost perfectly duopolistic system like the United States. Furthermore, it is not really possible to know if ANES respondents were "hopeful" or not about their candidate as no measure is available to distinguish optimistic from pessimistic voters. We only know about their expectations. This constitutes one of the limitations of the present paper. However, in the absence of adequate measures, the role of voters' optimism toward parties' or candidates' faith will have to be investigated by future research.

– *1996 U.S. presidential election.* Who do you think will be elected President in November? Bill Clinton, Bob Dole, Ross Perot, or another candidate?

– *2000 U.S. presidential election.* Who do you think will be elected President in November? Al Gore, George W. Bush, Pat Buchanan, Ralph Nader, or another candidate?

- *2004 U.S. presidential election.* Who do you think will be elected President in November? John Kerry, George W. Bush, Ralph Nader, or another candidate?
- *2012 U.S. presidential election.* Who do you think will be elected President in November? Barack Obama, Mitt Romney, or another candidate?
- *2016 U.S. presidential election.* Who do you think will be elected President in November? Hillary Clinton, Donald Trump, or another candidate?
- *2020 U.S. presidential election.* Who do you think will be elected President in November? Joe Biden, Donald Trump, or another candidate?

Expected closeness. Respondents who believed the election would be close were coded 0, while those who believed the election would be won by quite a bit were coded 1.

- *1996–2020 U.S. presidential elections.* Do you think the presidential race will be close or will [predicted winner] win by quite a bit?

B.3 Electoral Status

Party identification. The electoral status of respondents was determined by using their party identification. Respondents were categorized either as "losers" (1), "independents/others" (2), or "winners" (3). Leaners were classified as losers or winners depending on the party they were leaning toward since leaners tend to have attitudes and behaviours that are very similar to those of partisans (Keith et al., 1992; Magleby, Nelson and Westlye, 2011).

- *1996–2020 U.S. presidential elections.* Generally speaking, do you usually think of yourself as a Democrat, a Republican, an independent, or what?

Reported vote. As an alternative, the electoral status of respondents was also determined using their reported vote. Respondents were categorized either as "losers" (1), "non-voters" (2), or "winners" (3).

- *1996 U.S. presidential election.* How about the election for President? Did you vote for a candidate for President? Yes, voted for president/No, didn't vote for President. [If yes] Who did you vote for? Bill Clinton (1), Bob Dole (2), Ross Perot (3), or another candidate (4)?
- *2000 U.S. presidential election.* How about the election for President? Did you vote for a candidate for President? Yes, voted for president/No, didn't vote for President. [If yes] Who did you vote for? Al Gore (1), George W. Bush (2), Pat Buchanan (3), Ralph Nader (4), or another candidate (5)?
- *2004 U.S. presidential election.* In talking to people about elections, we often find that a lot of people were not able to vote because they weren't registered, they were sick, or they just didn't have time. [How about you—did you vote in the elections this November?/Which of the following statements best describes you?] Yes, voted [I am sure I voted]/No, didn't vote [I did not vote (in the election this November)/I thought about voting this time, but didn't/I usually vote, but didn't this time]. [If yes] Who did you vote for? John Kerry (1), George W. Bush (2), Ralph Nader (3), or another candidate (4)?
- *2012 U.S. presidential election.* How about the election for President? Did you vote for a candidate for President? Yes, voted for President/No, didn't vote for President. [If yes] Who did you vote for? Barack Obama (1), Mitt Romney (2), or another candidate (3)?
- *2016 U.S. presidential election.* How about the election for President? Did you vote for a candidate for President? Yes, voted for President/No, didn't vote for President. [If yes] Who did you vote for? Hillary Clinton (1), Donald Trump (2), Gary Johnson (3), Jill Stein (4), or another candidate (5)?

– *2020 U.S. presidential election.* How about the election for President? Did you vote for a candidate for President? Yes, voted for President/No, didn't vote for President. [If yes] Who did you vote for? Joe Biden (1), Donald Trump (2), Jo Jorgensen (3), Howie Hawkins (4), or another candidate (5)?

Party identification strength. The strength of respondents' partisan identification was coded 0 for weak partisans and leaners and 1 for strong partisans.

– *1996–2020 U.S. presidential elections.* [If R considers self a Democrat/Republican] Would you call yourself a strong [Democrat/Republican] or a not very strong [Democrat/Republican]? [If no party identification] Do you think of yourself as closer to the Republican Party or to the Democratic Party?

B.4 Suspicion

Conspiracy beliefs. Answers given by respondents about potential conspiracies[2] were summated to form a conspiratorial thinking scale and the index was re-scaled to a 0–1 interval. Values of 0 indicate the absence of conspiratorial beliefs and values of 1 indicate the highest level of conspiratorial thinking. For the 2012 presidential election, the index includes respondents' beliefs about Barack Obama's birthplace, the "death panel" provision (i.e., whether or not the Affordable Care Act authorizes end-of-life decisions), government's knowledge of the 9/11 terror attacks beforehand, and intentional flooding of poor areas by the government to protect richer neighbourhoods during Hurricane Katrina. For the 2016 presidential election, the index includes respondents' beliefs about government's knowledge of 9/11 and Barack Obama's religion. For the 2020 presidential election, the conspiracy items are less specific (and perhaps less tainted by partisanship). Respondents were asked whether most business and politics in the U.S. were controlled by a few powerful people and whether much of what people hear in schools and media are lies designed to keep people from learning the real truth about those in power.

– *2012 U.S. presidential election.* (a) Was Barack Obama definitely born in the United States, probably born in the United States, probably born in another country, or definitely born in another country? (b) Does the health care law passed in 2010 definitely authorize government panels to make end-of-life decisions for people on Medicare, probably authorize government panels to make end-of-life decisions for people on Medicare, probably not authorize government panels to make end-of-life decisions for people on Medicare, or definitely not authorize government panels to make end-of-life decisions for people on Medicare? (c) Did senior federal government officials definitely know about the terrorist attacks on September 11, 2001 before they happened, probably knew about the terrorist attacks on September 11, 2001 before they happened, probably did not know about the terrorist attacks on September 11, 2001 before they happened, or definitely did not know about the terrorist attacks on September 11, 2001 before they happened? (d) Some people say that when Hurricane Katrina hit the Gulf Coast in

[2] The line between false or inaccurate beliefs and conspiracy theories is sometimes hard to trace. Only items implying that social or political actors were voluntarily hiding the truth or acting against the common good (with no evidence to that effect) were used to create the conspiratorial thinking scale. Therefore, opinions contrary to scientific consensus—e.g., vaccines lead to an increased risk of autism, global warming is probably not happening, etc.—were not used to measure conspiracism, although these ideas are most probably indicative of conspiratorial thinking. All conspiracy items were asked in the *post-election* wave of the 2012, 2016, and 2020 ANES. From a theoretical perspective, conspiratorial thinking should be causally prior to expectations. In 2012 and 2016, belief in conspiracy theories was assessed in relation to specific events or topics (none of them related to the integrity of elections or the functioning of democracy) such as U.S. officials' foreknowledge of the September 11 attacks or Barack Obama's birthplace. Therefore, there is no reason to believe that voters' expectations about election outcomes or the outcomes themselves could have had an influence on the beliefs of ANES respondents regarding the truthfulness of these conspiracy theories. Rather than measure respondents beliefs in specific conspiracy theories, the 2020 ANES includes broader indicators of conspiratorial *predispositions* or conspiratorial thinking. Although it is possible that the outcome of the 2020 election might have negatively skewed the answers of Trump supporters on these two items, conspiratorial thinking should be conceptualized as a relatively stable trait. According to Edelson et al. (2017, 936), "[j]ust as underlying political predispositions, such as left/right ideology or partisanship, shape how people form more specific opinions, evaluate information, and choose among alternatives, underlying conspiratorial thinking drives people to attach conspiratorial explanations to specific events and circumstances as they come to pass." Additionally, in a study conducted in the weeks *following* the 2016 presidential election, Lamberty, Hellmann and Oeberst (2018) found that conspiracy mentality (a scale made of multiple items with no reference to specific conspiracies or groups, e.g., "There are secret organizations that have great influence on political decisions") was more prevalent among Trump voters than it was among Clinton voters.

the summer of 2005, the federal government intentionally breached flood levees in New Orleans so that poor neighborhoods would be flooded and middle-class neighborhoods would be spared. Do you think the federal government definitely did this, probably did this, probably did not do this, or definitely did not do this?

– *2016 U.S. presidential election.* (a) Did senior federal government officials definitely know about the terrorist attacks on September 11, 2001 before they happened, probably know about the terrorist attacks on September 11, 2001 before they happened, probably not know about the terrorist attacks on September 11, 2001 before they happened, or definitely not know about the terrorist attacks on September 11, 2001 before they happened? (b) Is Barack Obama a Muslim, or is he not a Muslim?

– *2020 U.S. presidential election.* (a) How well does the following statement describe your view? "Most business and politics in this country are secretly controlled by the same few powerful people." Not at all, not very well, somewhat well, very well, or extremely well? (b) How well does the following statement describe your view? "Much of what people hear in schools and the media are lies designed to keep people from learning the real truth about those in power." Not at all, not very well, somewhat well, very well, or extremely well?

Political cynicism. Brants (2013, 16) has defined cynicism as "the absence of a belief in the reliability of authorities, or no or limited faith in their sincerity," while Miller (1974, 952) refers to it as "the degree of negative affect toward the government and [as] a statement of the belief that the government is not functioning and producing outputs in accord with individual expectations" (see also Kaid, McKinney and Tedesco 2000, 198). Defining and measuring political cynicism have proved to be difficult endeavours (Rijkhoff, 2018). In fact, political cynicism is often "equated to other concepts such as distrust, skepticism and a lack of efficacy" (Rijkhoff, 2018, 333). According to Rijkhoff (2018), six recurring determinants of political cynicism can be derived from existing studies, that is (1) untrustworthiness, (2) immorality, (3) dishonesty, (4) incompetence, (5) self-interest, and (6) being out of touch with citizens. Hence, many definitions of political cynicism overlap with definitions of trust and external efficacy.[3] Answers given by respondents on items measuring cynical attitudes were summated to form a political cynicism scale and the index was re-scaled to a 0–1 interval.

– *1996 U.S. presidential election.* [Pre-election wave] (a) People have different ideas about the government in Washington. These ideas don't refer to Democrats or Republicans in particular, but just to the government in general. We want to see how you feel about these ideas. For example: How much of the time do you think you can trust the government in Washington to do what is right? Just about always, most of the time, or only some of the time? [Post-election wave] (a) Some people say that members of Congress know what ordinary people think. Others say that members of Congress don't know much about what ordinary people think. Using the scale in the booklet, (where 1 means that the members of Congress know what ordinary people think, and 5 means that the members of don't know much about what ordinary people think), where would you place? (b) Please tell me how much you agree or disagree with these statements. The first is: "Public officials don't care much what people like me think." Do you agree strongly, agree somewhat, neither agree nor disagree, disagree somewhat, or disagree strongly with this statement? (c) Over the years, how much attention do you feel the government pays to what people think when it decides what to do? A good deal, some, or not much? (d) People have different ideas about the government in Washington. These ideas don't refer to Democrats or Republicans in particular, but just to the government in general. We want to see how you feel about these ideas. For example: How much of the time do you think you can trust the government in Washington to do what is right—just about always, most of the time, or only some of the time? Just about always, most of the time, only some of the time, or never (voluntary)? (e) Do you think that people in government waste a lot of the money we pay in taxes, waste some of it, or don't waste very much of it? (f) Would you say the government is pretty much run by a few big interests looking out for themselves or that it is run for the benefit of all the people? (g) Do you think that quite a few of the people running the government are crooked, not very many are, or do you think hardly any of them are crooked?

[3] Items related to *internal* political efficacy (e.g., "People like me don't have any say about what the government does" or "Sometimes politics and government seem so complicated that a person like me can't really understand what's going on") were thus excluded. Although the "people like me don't have any say" item is often treated as an external efficacy question, it may also be interpreted as a measure of perceived self-competence (Kornberg and Clarke, 1992).

- *2000 U.S. presidential election.* [Post-election wave] (a) Please tell me how much you agree or disagree with these statements about the government. The first is: "Public officials don't care much what people like me think." Do you agree strongly, agree somewhat, neither agree nor disagree, disagree somewhat, or disagree strongly with this statement? (b) Over the years, how much attention do you feel the government pays to what people think when it decides what to do? A good deal, some, or not much? (c) People have different ideas about the government in Washington. These ideas don't refer to Democrats or Republicans in particular, but just to the government in general. We want to see how you feel about these ideas. For example: How much of the time do you think you can trust the government in Washington to do what is right? Just about always, most of the time, or only some of the time? (d) Do you think that people in government waste a lot of the money we pay in taxes, waste some of it, or don't waste very much of it? (e) Would you say the government is pretty much run by a few big interests looking out for themselves or that it is run for the benefit of all the people? (f) Do you think that quite a few of the people running the government are crooked, not very many are, or do you think hardly any of them are crooked?

- *2004 U.S. presidential election.* [Post-election wave] (a) I'd like to read you a few statements about public life. I'll read them one at a time and please tell me how strongly you agree or disagree with each of them. "Public officials don't care much what people like me think." Do you agree strongly, agree somewhat, neither agree nor disagree, disagree somewhat, or disagree strongly with this statement? (b) Over the years, how much attention do you feel the government pays to what people think when it decides what to do? A good deal, some, or not much? (c) People have different ideas about the government in Washington. These ideas don't refer to Democrats or Republicans in particular, but just to the government in general. We want to see how you feel about these ideas. For example: How much of the time do you think you can trust the government in Washington to do what is right? Just about always, most of the time, or only some of the time? (d) Do you think that people in government waste a lot of the money we pay in taxes, waste some of it, or don't waste very much of it? (e) Would you say the government is pretty much run by a few big interests looking out for themselves or that it is run for the benefit of all the people? (f) Do you think that quite a few of the people running the government are crooked, not very many are, or do you think hardly any of them are crooked? (g) Some people say it makes a difference who is in power. Others say that it doesn't make a difference who is in power. Using the scale in the booklet, (where one means that it makes a difference who is in power and five means that it doesn't make a difference who is in power), where would you place yourself?

- *2012 U.S. presidential election.* [Pre-election wave] (a) How often can you trust the federal government in Washington to do what is right? Always, most of the time, about half the time, some of the time, or never? (b) Would you say the government is pretty much run by a few big interests looking out for themselves or that it is run for the benefit of all the people? (c) Do you think that people in government waste a lot of the money we pay in taxes, waste some of it, or don't waste very much of it? (d) How many of the people running the government are corrupt? All, most, about half, a few, or none? [Post-election wave] (a) Do you think that quite a few of the people running the government are crooked, not very many are, or do you think hardly any of them are crooked? (b) "Public officials don't care much what people like me think." Do you agree strongly, agree somewhat, neither agree nor disagree, disagree somewhat, or disagree strongly? (c) Some people say that it doesn't make any difference who is in power. Others say that it makes a big difference who is in power. Using the scale in the booklet, (where one means that it doesn't make any difference who is in power and five means that it makes a big difference who is in power), where would you place yourself?

- *2016 U.S. presidential election.* [Pre-election wave] (a) How often can you trust the federal government in Washington to do what is right? Always, most of the time, about half the time, some of the time, or never? (b) Would you say the government is pretty much run by a few big interests looking out for themselves or that it is run for the benefit of all the people? (c) Do you think that people in government waste a lot of the money we pay in taxes, waste some of it, or don't waste very much of it? (d) How many of the people running the government are corrupt? All, most, about half, a few, or none? [Post-election wave] (a) "Public officials don't care much what people like me think." Do you agree strongly, agree somewhat, neither agree nor disagree, disagree somewhat, or disagree strongly?

(b) How widespread do you think corruption such as bribe taking is among politicians in the United States? Very widespread, quite widespread, not very widespread, or it hardly happens at all? (c) "Most politicians do not care about the people." Do you agree strongly, agree somewhat, neither agree nor disagree, disagree somewhat, or disagree strongly? (d) "Most politicians are trustworthy." Do you agree strongly, agree somewhat, neither agree nor disagree, disagree somewhat, or disagree strongly? (e) "Politicians are the main problem in the United States." Do you agree strongly, agree somewhat, neither agree nor disagree, disagree somewhat, or disagree strongly? (f) "Most politicians care only about the interests of the rich and powerful." Do you agree strongly, agree somewhat, neither agree nor disagree, disagree somewhat, or disagree strongly?

- *2020 U.S. presidential election.* [Pre-election wave] (a) Do you think that people in government waste a lot of the money we pay in taxes, waste some of it, or don't waste very much of it? (b) Would you say the government is pretty much run by a few big interests looking out for themselves or that it is run for the benefit of all the people? (c) How many of the people running the government are corrupt? All, most, about half, a few, or none? [Post-election wave] (a) "Public officials don't care much what people like me think." Do you agree strongly, agree somewhat, neither agree nor disagree, disagree somewhat, or disagree strongly? (b) How widespread do you think corruption such as bribe taking is among politicians in the United States? Very widespread, quite widespread, not very widespread, or it hardly happens at all? (c) "Most politicians do not care about the people." Do you agree strongly, agree somewhat, neither agree nor disagree, disagree somewhat, or disagree strongly? (d) "Most politicians are trustworthy." Do you agree strongly, agree somewhat, neither agree nor disagree, disagree somewhat, or disagree strongly? (e) "Politicians are the main problem in the United States." Do you agree strongly, agree somewhat, neither agree nor disagree, disagree somewhat, or disagree strongly? (f) "Most politicians care only about the interests of the rich and powerful." Do you agree strongly, agree somewhat, neither agree nor disagree, disagree somewhat, or disagree strongly?

Social mistrust. Social mistrust was measured on a four-point scale for the 2012–2020 U.S. presidential election. The variable was re-scaled to a 0–1 interval. For the 1996–2004 U.S. presidential election, Social mistrust was a binary variable coded 0 for respondents who believed most people could be trusted and 1 for those you thought that you could not be too careful in dealing with people.[4]

- *1996–2004 U.S. presidential elections.* Generally speaking, would you say that most people can be trusted, or that you can't be too careful in dealing with people?
- *2012–2020 U.S. presidential elections.* Generally speaking, how often can you trust other people? Always, most of the time, about half the time, some of the time, or never?

B.5 Economic Evaluations

Egotropic evaluations of the economy. Respondents were asked about the state of their personal financial situation at different points in time. Answers were summated to form an egotropic economic evaluations index and the index was re-scaled to a 0–1 interval.

- *1996–2020 U.S. presidential elections.* (a) We are interested in how people are getting along financially these days. Would you say that you (and your family living here) are much better off financially, somewhat better off, about the same, somewhat worse off, or much worse off than you were a year ago? [If better] Is that much better off or somewhat better off? [If worse] Is that much worse off or somewhat worse off? (b) Now looking ahead, do you think that a year from now you (and your family living here) will be much better off financially, somewhat better off, about the same, somewhat worse off, or much worse off than now? [If better] Is that much better off or somewhat better off? [If worse] Is that much worse off or somewhat worse off?

[4] The social mistrust item was asked in the post-election wave survey of the 2000 and 2004 ANES.

Sociotropic evaluations of the economy. Respondents were asked about the state of U.S. economy at different points in time. Answers were summated to form a sociotropic economic evaluations index and the index was re-scaled to a 0–1 interval.

- *1996–2020 U.S. presidential elections.* (a) Now thinking about the economy in the country as a whole, would you say that over the past year the nation's economy has gotten better, stayed about the same, or gotten worse? [If better] Would you say much better or somewhat better? [If worse] Would you say much worse or somewhat worse? (b) What about the next 12 months? Do you expect the economy, in the country as a whole, to get better, stay about the same, or get worse? [If better] Would you say much better or somewhat better? [If worse] Would you say much worse or somewhat worse?

B.6 Political Knowledge

Political knowledge. Political knowledge items were coded as either "correct" (1) or "incorrect" (0). "Don't know" answers were treated as incorrect. Answers were summated to form a knowledge scale and the index was re-scaled to a 0–1 interval.

- *1996 U.S. presidential election.* (a) Now we have a set of questions concerning various public figures. We want to see how much information about them gets out to the public from television, newspapers and the like. The first name is Al Gore. What job or political office does he now hold? Correct answer: Vice-President. (b) William Rehnquist? Correct answer: Chief Justice of the Supreme Court. (c) Boris Yeltsin? Correct answer: President of Russia or leader/head/top man of Russia. (d) Newt Gingrich? Correct answer: Speaker of the House of Representatives. (e) Do you happen to know which party had the most members in the House of Representatives in Washington before the election [this/last] month? Correct answer: Republican Party. (f) Do you happen to know which party had the most members in the U.S. Senate before the election [this/last] month? Correct answer: Republican Party.

- *2000 U.S. presidential election.* (a) Now we have a set of questions concerning various public figures. We want to see how much information about them gets out to the public from television, newspapers and the like. The first name is Trent Lott. What job or political office does he now hold? Correct answer: Senate majority leader. (b) William Rehnquist? Correct answer: Chief Justice of the Supreme Court. (c) Tony Blair? Correct answer: Prime Minister of England/Great Britain. (d) Janet Reno? Correct answer: Attorney General of the United States. (e) Next, I'd like to ask you about the candidates who ran for President and their running mates. We're interested in some of the things that people may have heard about these candidates. The first candidate I'd like to ask you about is George W. Bush. What U.S. state does George W. Bush live in now? Correct answer: Texas. (f) What is George W. Bush's religion? Correct answer: Methodist. (g) Now take Al Gore. What U.S. state is Al Gore from originally? Correct answer: Tennessee. (h) What is Al Gore's religion? Correct answer: Baptist. (i) What about Dick Cheney. What U.S. state does Dick Cheney live in now? Correct answer: Wyoming. (j) What is Dick Cheney's religion? Correct answer: Methodist. (k) And Joseph Lieberman. What U.S. state does Joseph Lieberman live in now? Correct answer: Connecticut. (l) What is Joseph Lieberman's religion? Correct answer: Jewish. (m) Do you happen to know which party had the most members in the House of Representatives in Washington before the election [this/last] month? Correct answer: Republican Party. (n) Do you happen to know which party had the most members in the U.S. Senate before the election [this/last] month? Correct answer: Republican Party.

- *2004 U.S. presidential election.* (a) Now we have a set of questions concerning various public figures. We want to see how much information about them gets out to the public from television, newspapers and the like. The first name is Dennis Hastert. What job or political office does he now hold? Correct answer: Speaker of the House of Representatives. (b) Dick Cheney? Correct answer: Vice-President. (c) Tony Blair? Correct answer: Prime Minister of England/Great Britain. (d) William Rehnquist? Correct answer: Chief Justice of the Supreme Court. (e) Do you happen to know which party had the most members in the House of Representatives in Washington before the election [this/last] month? Correct answer: Republican Party. (f) Do you happen to know which party had the most members in the U.S. Senate before the election [this/last] month? Correct answer: Republican Party.

- *2012 U.S. presidential election.* (a) Now we have a set of questions concerning various public figures. We want to see how much information about them gets out to the public from television, newspapers and the like. The first name is John Boehner. What job or political office does he now hold? Correct answer: Speaker of the House of Representatives. (b) Joe Biden? Correct answer: Vice-President. (c) David Cameron? Correct answer: Prime Minister of UK. (d) John Roberts? Correct answer: Chief Justice of the Supreme Court. (e) Do you happen to know which party had the most members in the House of Representatives in Washington before the election [this/last] month? Correct answer: Republican Party. (f) Do you happen to know which party had the most members in the U.S. Senate before the election [this/last] month? Correct answer: Democratic Party.

- *2016 U.S. presidential election.* (a) Now we have a set of questions concerning various public figures. We want to see how much information about them gets out to the public from television, newspapers and the like. The first name is Joe Biden. What job or political office does he now hold? Correct answer: Vice-President. (b) Paul Ryan? Correct answer: Speaker of the House of Representatives. (c) Angela Merkel? Correct answer: German Chancellor. (d) Vladimir Putin? Correct answer: Russian President. (e) John Roberts? Correct answer: Chief Justice of the Supreme Court. (f) Do you happen to know which party currently has the most members in the House of Representatives in Washington? Correct answer: Republican Party. (g) Do you happen to know which party currently has the most members in the U.S. Senate? Correct answer: Republican Party.

- *2020 U.S. presidential election.*[5] (a) For how many years is a United States Senator elected—that is, how many years are there in one full term of office for a U.S. Senator? Correct answer: Six years. (b) On which of the following does the U.S. federal government currently spend the least? Correct answer: Foreign aid. (c) Do you happen to know which party currently has the most members in the House of Representatives in Washington? Correct answer: Democratic Party. (d) Do you happen to know which party currently has the most members in the U.S. Senate? Correct answer: Republican Party. (e) Now we have a set of questions concerning various public figures. The first name is Mike Pence. What job or political office does he now hold? Correct answer: Vice-President. (f) Nancy Pelosi? Correct answer: Speaker of the House of Representatives. (g) Angela Merkel? Correct answer: German Chancellor. (h) Vladimir Putin? Correct answer: Russian President. (i) John Roberts? Correct answer: Chief Justice of the Supreme Court.

B.7 Sociodemographics

Age. Age is the respondents' age in years.

Education. Education is an ordinal variable that ranges from "8 grades or less" (1) to "advanced degree, including LLB" (7) in the 1996–2004 ANES, and from "less than 1st grade" (1) to "doctorate degree (for example: PhD, EdD)" (16) in the 2012–2020 ANES. In 1996, 2000, and 2004, education is a composite of three related items.

- *1996–2004 U.S. presidential elections.* What is the highest grade of school or year of college you have completed? (0–16) 0–16 grades, (17) 17 grades or more. [If highest grade of education is 0-12 years or DK] Did you get a high school diploma or pass a high school equivalency test? (1) yes, (5) no. [If highest grade of education is 13+ years] What is the highest degree that you have earned? (1) bachelor's degree, (2) master's degree, (3) PhD, LIT, SCD, DFA, DLIT, DPH, DPHIL, JSC, SJD, (4) LLB, JD, (5) MD, DDS, DVM, MVSA, DSC, DO, (6) JDC, STD, THD, (7) associate degree (AA), (0/96) no degree earned.

- *2012–2020 U.S. presidential elections.* What is the highest level of school you have completed or the highest degree you have received? (1) less than 1st grade, (2) 1st, 2nd, 3rd or 4th grade, (3) 5th or 6th grade, (4) 7th or 8th grade, (5) 9th grade, (6) 10th grade, (7) 11th grade, (8) 12th grade no diploma, (9) high school graduate – high school diploma or equivalent (for example: GED), (10) some college but

[5] Catch questions were excluded.

no degree, (11) associate degree in college – occupational/vocational program, (12) associate degree in college – academic program, (13) bachelor's degree (for example: BA, AB, BS), (14) master's degree (for example: MA, MS, MEng, MEd, MSW, MBA), (15) professional school degree (for example: MD, DDS, DVM, LLB, JD), (16) doctorate degree (for example: PhD, EdD), (95) other.

Gender. Gender is a binary variable coded 0 for female respondents and 1 for male respondents.

Household income. Household income was measured on varying scales depending on the survey.

- *1996 U.S. presidential election.* Please look at the booklet and tell me the letter of the income group that includes the income of all members of your family living here in 1995 before taxes. This figure should include salaries, wages, pensions, dividends, interest, and all other income. (1) none or less than $2,999, (2) $3,000–$4,999, (3) $5,000–$6,999, (4) $7,000–$8,999, (5) $9,000–$9,999, (6) $10,000–$10,999, (7) $11,000–$11,999, (8), $12,000–$12,999, (9) $13,000–$13,999, (10) $14,000–$14.999, (11) $15,000–$16,999, (12) $17,000–$19,999, (13) $20,000–$21,999, (14) $22,000–$24,999, (15) $25,000–$29,999, (16) $30,000–$34,999, (17) $35,000–$39,999, (18) $40,000–$44,999, (19) $45,000–$49,999, (20) $50,000–$59,999, (21) $60,000–$74,999, (22) $75,000–$89,999, (23) $90,000–$104,999, (24) $105,000 and over.
- *2000 U.S. presidential election.* Please look at the booklet and tell me the letter of the income group that includes the income of all members of your family living here in 1999 before taxes. This figure should include salaries, wages, pensions, dividends, interest, and all other income. (1) None or less than $4,999, (2) $5,000–$9,999, (3) $10,000–$14,999, (4) $15,000–$24,999, (5) $25,000–$34,999, (6) $35,000–$49,999, (7) $50,000–$64,999, (8) $65,000–$74,999, (9) $75,000–$84,999, (10) $85,000–$94,999, (11) $95,000–$104,999, (12) $105,000–$114,999, (13) $115,000–$124,999, (14) $125,000–$134,999, (15) $135,000–$144,999, (16) $145,000–$154,999, (17) $155,000–$164,999, (18) $165,000–$174,999, (19) $175,000–$184,999, (20) $185,000–$199,999, (21) $200,000 and over.
- *2004 U.S. presidential election.* Please look at the booklet and tell me the letter of the income group that includes the income of all members of your family living here in 2003 before taxes. This figure should include salaries, wages, pensions, dividends, interest, and all other income. (1) none or less than $2,999, (2) $3,000–$4,999, (3) $5,000–$6,999, (4) $7,000–$8,999, (5) $9,000–$10,999, (6) $11,000–$12,999, (7) $13,000–$14,999, (8) $15,000–$16,999, (9) $17,000–$19,999, (10) $20,000–$21,999, (11) $22,000–$24,999, (12) $25,000–$29,999, (13) $30,000–$34,999, (14) $35,000–$39,999, (15) $40,000–$44,999, (16) $45,000–$49,999, (17) $50,000–$59,999, (18) $60,000–$69,999, (19) $70,000–$79,999, (20) $80,000–$89,999, (21) $90,000–$104,999, (22) $105,000–$119,000, (23) $120,000 and over.
- *2012 U.S. presidential election.* The next question is about the total income of all the members of your family living here in 2011, before taxes. This figure should include income from all sources, including salaries, wages, pensions, Social Security, dividends, interest, and all other income. What was the total income in 2011 of all your family members living here? (1) under $5,000, (2) $5,000–$9,999, (3) $10,000–$12,499, (4) $12,500–$14,999, (5) $15,000–$17,499, (6) $17,500–$19,999, (7) $20,000–$22,499, (8) $22,500–$24,999, (9) $25,000–$27,499, (10) $27,500–$29,999, (11) $30,000–$34,999, (12) $35,000–$39,999, (13) $40,000–$44,999, (14) $45,000–$49,999, (15) $50,000–$54,999, (16) $55,000–$59,999, (17) $60,000–$64,999, (18) $65,000–$69,999, (19) $70,000–$74,999, (20) $75,000–$79,999, (21) $80,000–$89,999, (22) $90,000–$99,999, (23) $100,000–$109,999, (24) $110,000–$124,999, (25) $125,000–$149,999, (26) $150,000–$174,999, (27) $175,000–$249,999, (28) $250,000 or more.
- *2016 U.S. presidential election.* The next question is about the total income of all the members of your family living here in 2015, before taxes. This figure should include income from all sources, including salaries, wages, pensions, Social Security, dividends, interest, and all other income. What was the total income in 2015 of all your family members living here? (1) under $5,000, (2) $5,000–$9,999, (3) $10,000–$12,499, (4) $12,500–$14,999, (5) $15,000–$17,499, (6) $17,500–$19,999, (7) $20,000–$22,499, (8) $22,500–$24,999, (9) $25,000–$27,499, (10) $27,500–$29,999, (11) $30,000–$34,999, (12) $35,000–$39,999, (13) $40,000–$44,999, (14) $45,000–$49,999, (15) $50,000–$54,999, (16) $55,000–$59,999, (17) $60,000–$64,999, (18) $65,000–$69,999, (19) $70,000–$74,999, (20) $75,000–$79,999,

(21) \$80,000–\$89,999, (22) \$90,000–\$99,999, (23) \$100,000–\$109,999, (24) \$110,000–\$124,999, (25) \$125,000–\$149,999, (26) \$150,000–\$174,999, (27) \$175,000–\$249,999, (28) \$250,000 or more.

– *2020 U.S. presidential election.* The next question is about the total combined income of all members of your family during the past 12 months. This includes money from jobs, net income from business, farm or rent, pensions, dividends, interest, Social Security payments, and any other money income received by members of your family who are 15 years of age or older. What was the total income of your family during the past 12 months? (1) under \$9,999, (2) \$10,000–\$14,999, (3) \$15,000–\$19,999, (4) \$20,000–\$24,999, (5) \$25,000–\$29,999, (6) \$30,000–\$34,999, (7) \$35,000–\$39,999, (8) \$40,000–\$44,999, (9) \$45,000–\$49,999, (10) \$50,000–\$59,999, (11) \$60,000–\$64,999, (12) \$65,000–\$69,999, (13) \$70,000–\$74,999, (14) \$75,000–\$79,999, (15) \$80,000–\$89,999, (16) \$90,000–\$99,999, (17) \$100,000–\$109,999, (18) \$110,000–\$124,999, (19) \$125,000–\$149,999, (20) \$150,000–\$174,999, (21) \$175,000–\$249,999, (22) \$250,000 or more.

Race. Race is a binary variable coded 1 if a respondent identifies as white and 0 otherwise.

Region fixed effects. Four regional dummies were created, one for each census region (i.e., Midwest, Northeast, South, and West).

B.8 *Descriptive Statistics*

Figure B.8.1 displays the distribution of responses for the two dependent variables among all ANES respondents for each election. More precisely, panel (a) shows the distribution of respondents across the four categories of the perceived electoral integrity variable. The vast majority of respondents seem to express confidence in the U.S. electoral process, although a non-negligible minority of individuals are doubtful about the fairness of elections. Note that the percentage of ANES respondents mentioning an unfair election is particularly high in 2000 (almost 45 per cent), which can probably be explained by the legal aftermath of the 2000 election and the controversy surrounding the Florida recount. It is important to mention that the distributions for the 1996–2004 elections cannot be easily compared with those for the 2012–2020 elections as different questions were used in the ANES to measure beliefs about electoral integrity. As mentioned above, while respondents were asked about the fairness of the last election in 1996–2004, respondents in the most recent ANES were questioned about the fairness of the vote count in *all* previous elections. This might partly explain why the proportion of respondents expressing doubts in the electoral process is relatively unimpressive in both 2016 and 2020 considering the highly contentious nature of these elections. Panel (b) shows the distribution of respondents across the four levels of satisfaction with democracy. We can see a decreasing trend among those being "satisfied" with the way democracy works, while the proportion of individuals stating that they are "not very satisfied" appears to be on the rise. The percentage of Democratic partisans not very or not at all satisfied with democracy has slightly increased between 2016 and 2020 (+4.2 percentage points, from 37 per cent to 41.2 per cent). A larger increase occurred among Republican supporters (+10.1 percentage points, from 25.5 per cent to 35.6 per cent). While the increase among Republicans can be explained, at the very least partially, by Biden's victory, it is somewhat more puzzling among Democrats. However, Fahey, Allen and Alarian (2022) have demonstrated that the success of right-wing populists—with which Trump share a number of characteristics (Rudolph, 2021; Schneiker, 2020)—is capable of durably decreasing democratic satisfaction among the mainstream electorate.

Figure B.8.1. Distribution of Responses for Each of the Dependent Variables, 1996–2020

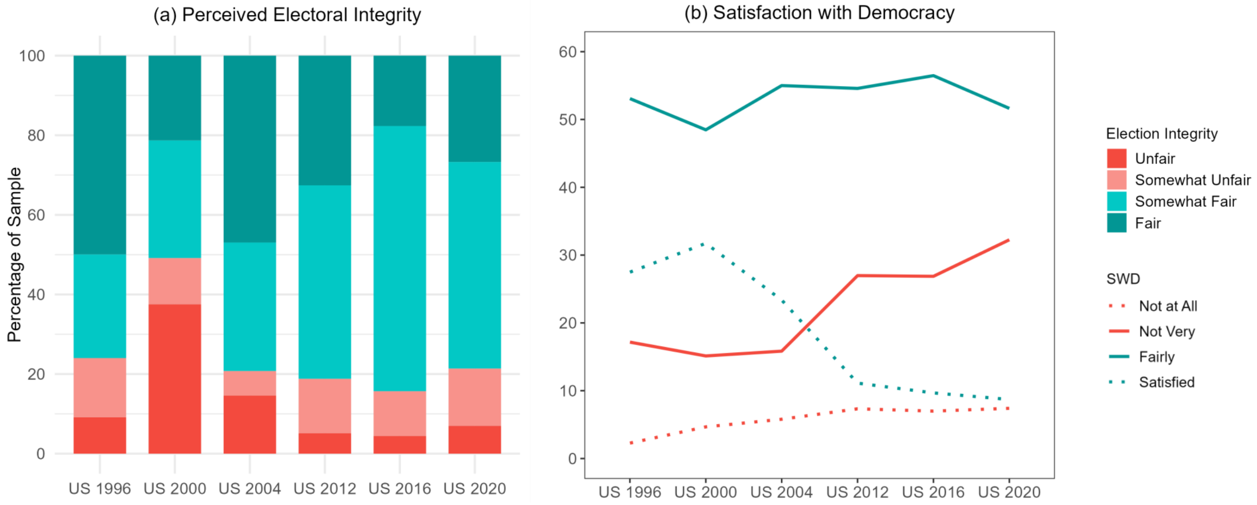

Note. Although the answer labels are not the same for perceived electoral integrity across surveys, they were relabelled here as "unfair," "somewhat unfair," "somewhat fair," and "fair" for the sake of simplification.

Panels (a) and (b) in Figure B.8.2 show the distribution of ANES respondents according to their party identification status and reported vote status, respectively. Panels (c) and (d) show the percentage of correct forecasts (expectations) by respondents' party identification status and reported vote status, respectively. Unsurprisingly, winners are much more likely to correctly predict the outcome than losers, a result that is in part attributable to wishful thinking and motivated reasoning. Note that pure independents and non-voters

are always very close to the mean percentage of correct forecasts in every election (as indicated by the black line). There are few differences between panels (c) and (d) as one could expect from the generally strong correlation between party identification and vote choice. Finally, panels (e) and (f) present density plots for the suspicion index according to respondents' level of perceived electoral integrity and satisfaction with democracy, respectively. As we could reasonably expect, the distribution is slightly skewed to the right of the suspicion index scale among respondents questioning the fairness of elections and those who are unsatisfied with the way democracy works in the U.S.

Figure B.8.2. Descriptive Statistics

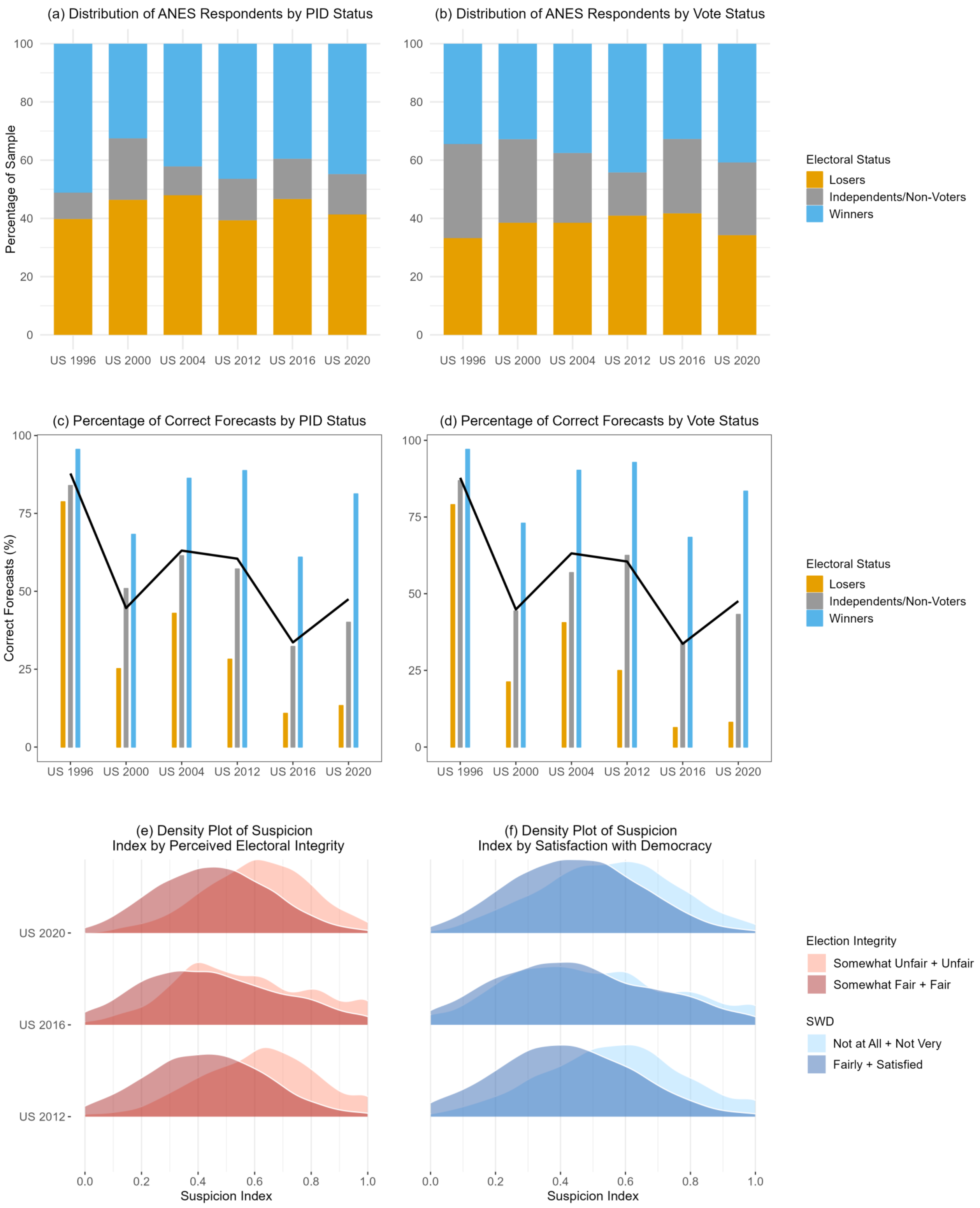

Note. The black line in panels (c) and (d) shows the average percentage of correct forecasts in each election.

C Regression Tables: 2-Way Interaction

The 2012, 2016, and 2020 models include controls for conspiracy beliefs, political cynicism, and social mistrust. In general, measures of suspicious thinking were negatively related to perceived electoral integrity and satisfaction with democracy. Attitudes conducive to suspicion are perhaps even more consequential than partisan biases in shaping voters' expectations; at the same time, feelings of mistrust are themselves often coloured by partisan attachments (e.g., Blais, Gidengil and Kilibarda 2017; Uscinski, Klofstad and Atkinson 2016). In 2012, both conspiracy beliefs and political cynicism had a substantial negative influence of perceived electoral integrity (-1.07 points and -0.70 points, respectively, when moving from the minimum to the maximum value of each scale) and satisfaction with democracy (-0.46 points and -1.13 points, respectively). In 2016, the impact of conspiracy beliefs was much less important, while cynicism still had a considerable influence on perceptions of fairness and democratic satisfaction (around -0.60 points in each case). In 2020, conspiratorial thinking was negatively related to both dependent variables (-0.58 points and -0.45 points, respectively), while political cynicism only had an impact on democratic satisfaction (-0.49 points). Across all three elections, social mistrust generally had a negative influence, but its impact appeared much more modest. See tables C.1.4 to C.1.6 (party identification) and tables C.2.4 to C.2.6 (reported vote).

C.1 Party Identification

Table C.1.1. Perceived Electoral Integrity and Satisfaction with Democracy, 1996 U.S. Presidential Election

	Winner-Loser Gap		Expectations	
	**Model 1.1** FAIR	**Model 2.1** SWD	**Model 1.2** FAIR	**Model 2.2** SWD
Electoral status				
Loser (R)				
Independent	-0.05	-0.11	-0.04	-0.14^{+}
Winner	0.26***	0.01	0.23***	0.01
Unexpected outcome?				
Yes			-0.14	0.02
2-way interaction				
Independent × Unexpected			-0.24	0.16
Winner × Unexpected			0.06	0.16
Economic evaluations				
Egotropic		0.26*		0.26*
Sociotropic		0.60***		0.60***
Information				
Political knowledge	0.43**	0.42***	0.43**	0.44***
Constant	2.45***	1.98***	2.50***	1.96***
Sociodemographics	Yes	Yes	Yes	Yes
Region fixed effects	Yes	Yes	Yes	Yes
Observations	1,367	1,329	1,363	1,325
R^2	0.06	0.11	0.06	0.11

Note. Ordinary Least Squares regressions. Significance levels: + $p < 0.10$; * $p < 0.05$; ** $p < 0.01$; *** $p < 0.001$. FAIR = Perceived electoral integrity. SWD = Satisfaction with democracy. R = Reference category.

Table C.1.2. Perceived Electoral Integrity and Satisfaction with Democracy, 2000 U.S. Presidential Election

	Winner-Loser Gap		Expectations	
	**Model 1.1** FAIR	**Model 2.1** SWD	**Model 1.2** FAIR	**Model 2.2** SWD
Electoral status				
Loser (R)				
Independent	-0.09	-0.41*	-0.29	-0.54*
Winner	0.45***	0.04	0.29	0.05
Unexpected outcome?				
Yes			-0.17	0.01
2-way interaction				
Independent × Unexpected			0.35	0.28
Winner × Unexpected			0.30	-0.01
Economic evaluations				
Egotropic		-0.16		-0.15
Sociotropic		0.77**		0.76**
Information				
Political knowledge	0.21	0.70**	0.22	0.66**
Constant	0.93***	2.03***	1.04***	2.04***
Sociodemographics	Yes	Yes	Yes	Yes
Region fixed effects	Yes	Yes	Yes	Yes
Observations	624	294	624	294
R^2	0.14	0.18	0.14	0.18

Note. Ordinary Least Squares regressions. Significance levels: + $p < 0.10$; * $p < 0.05$; ** $p < 0.01$; *** $p < 0.001$. FAIR = Perceived electoral integrity. SWD = Satisfaction with democracy. R = Reference category.

Table C.1.3. Perceived Electoral Integrity and Satisfaction with Democracy, 2004 U.S. Presidential Election

	Winner-Loser Gap		Expectations	
	**Model 1.1** FAIR	**Model 2.1** SWD	**Model 1.2** FAIR	**Model 2.2** SWD
Electoral status				
Loser (R)				
Independent	0.02	0.08	-0.03	-0.01
Winner	0.74***	0.33***	0.62***	0.25***
Unexpected outcome?				
Yes			-0.42***	-0.23**
2-way interaction				
Independent × Unexpected			-0.04	0.17
Winner × Unexpected			-0.11	0.14
Economic evaluations				
Egotropic		0.22		0.21
Sociotropic		0.78***		0.76***
Information				
Political knowledge	0.06	0.19^{+}	0.02	0.16
Constant	1.90***	1.92***	2.25***	2.10***
Sociodemographics	Yes	Yes	Yes	Yes
Region fixed effects	Yes	Yes	Yes	Yes
Observations	927	862	926	861
R^2	0.20	0.20	0.23	0.21

Note. Ordinary Least Squares regressions. Significance levels: + $p < 0.10$; * $p < 0.05$; ** $p < 0.01$; *** $p < 0.001$. FAIR = Perceived electoral integrity. SWD = Satisfaction with democracy. R = Reference category.

Table C.1.4. Perceived Electoral Integrity and Satisfaction with Democracy, 2012 U.S. Presidential Election

	Winner-Loser Gap		Expectations	
	**Model 1.1** FAIR	**Model 2.1** SWD	**Model 1.2** FAIR	**Model 2.2** SWD
Electoral status				
Loser (R)				
Independent	0.01	-0.14^{**}	-0.04	-0.06
Winner	0.07^{+}	-0.00	0.00	0.02
Unexpected outcome?				
Yes			-0.13^{**}	0.02
2-way interaction				
Independent × Unexpected			0.06	-0.18^{+}
Winner × Unexpected			-0.02	-0.08
Suspicious thinking				
Conspiracy beliefs	-1.13^{***}	-0.47^{***}	-1.07^{***}	-0.46^{***}
Political cynicism	-0.72^{***}	-1.12^{***}	-0.70^{***}	-1.13^{***}
Social mistrust	-0.05^{**}	-0.04^{*}	-0.05^{**}	-0.04^{*}
Economic evaluations				
Egotropic		0.17^{*}		0.17^{*}
Sociotropic		0.37^{***}		0.35^{**}
Information				
Political knowledge	-0.03	-0.11^{+}	-0.02	-0.11^{+}
Constant	3.48^{***}	3.44^{***}	3.54^{***}	3.43^{***}
Sociodemographics	Yes	Yes	Yes	Yes
Region fixed effects	Yes	Yes	Yes	Yes
Observations	4,444	4,330	4,424	4,314
R^2	0.19	0.17	0.19	0.17

Note. Ordinary Least Squares regressions. Significance levels: + $p < 0.10$; * $p < 0.05$; ** $p < 0.01$; *** $p < 0.001$. FAIR = Perceived electoral integrity. SWD = Satisfaction with democracy. R = Reference category.

Table C.1.5. Perceived Electoral Integrity and Satisfaction with Democracy, 2016 U.S. Presidential Election

	Winner-Loser Gap		Expectations	
	**Model 1.1** FAIR	**Model 2.1** SWD	**Model 1.2** FAIR	**Model 2.2** SWD
Electoral status				
Loser (R)				
Independent	-0.06	-0.03	0.04	-0.08
Winner	0.11^{**}	0.27^{***}	0.04	0.19^{*}
Unexpected outcome?				
Yes			-0.10	-0.11
2-way interaction				
Independent × Unexpected			-0.17	0.05
Winner × Unexpected			0.10	0.09
Suspicious thinking				
Conspiracy beliefs	-0.16^{*}	0.07	-0.18^{**}	0.05
Political cynicism	-0.55^{***}	-0.61^{***}	-0.59^{***}	-0.63^{***}
Social mistrust	-0.08^{***}	-0.02	-0.08^{***}	-0.02
Economic evaluations				
Egotropic		-0.10		-0.10
Sociotropic		0.50^{***}		0.52^{***}
Information				
Political knowledge	0.22^{**}	0.10	0.23^{**}	0.11
Constant	3.04^{***}	2.57^{***}	3.14^{***}	2.66^{***}
Sociodemographics	Yes	Yes	Yes	Yes
Region fixed effects	Yes	Yes	Yes	Yes
Observations	3,103	3,047	3,098	3,042
R^2	0.13	0.11	0.14	0.11

Note. Ordinary Least Squares regressions. Significance levels: + $p < 0.10$; * $p < 0.05$; ** $p < 0.01$; *** $p < 0.001$. FAIR = Perceived electoral integrity. SWD = Satisfaction with democracy. R = Reference category.

Table C.1.6. Perceived Electoral Integrity and Satisfaction with Democracy, 2020 U.S. Presidential Election

	Winner-Loser Gap		Expectations	
	**Model 1.1** FAIR	**Model 2.1** SWD	**Model 1.2** FAIR	**Model 2.2** SWD
Electoral status				
Loser (R)				
Independent	0.30^{***}	-0.15^{**}	0.07	-0.17^{*}
Winner	0.62^{***}	-0.05	0.26^{***}	-0.09
Unexpected outcome?				
Yes			-0.51^{***}	-0.08
2-way interaction				
Independent × Unexpected			0.18^{+}	0.01
Winner × Unexpected			0.21^{**}	-0.00
Suspicious thinking				
Conspiracy beliefs	-0.68^{***}	-0.47^{***}	-0.58^{***}	-0.45^{***}
Political cynicism	-0.06	-0.50^{***}	-0.07	-0.49^{***}
Social mistrust	-0.07^{***}	-0.01	-0.06^{***}	-0.01
Economic evaluations				
Egotropic		0.06		0.06
Sociotropic		0.27^{***}		0.29^{***}
Information				
Political knowledge	0.40^{***}	-0.13^{+}	0.36^{***}	-0.14^{+}
Constant	2.69^{***}	2.92^{***}	3.10^{***}	2.96^{***}
Sociodemographics	Yes	Yes	Yes	Yes
Region fixed effects	Yes	Yes	Yes	Yes
Observations	6,571	6,497	6,543	6,474
R^2	0.26	0.08	0.30	0.08

Note. Ordinary Least Squares regressions. Significance levels: + $p < 0.10$; * $p < 0.05$; ** $p < 0.01$; *** $p < 0.001$. FAIR = Perceived electoral integrity. SWD = Satisfaction with democracy. R = Reference category.

Table C.1.7. Perceived Electoral Integrity (Pre/Post), 2020 U.S. Presidential Election

	**Model 1**<br>No controls	**Model 2**<br>Controls
Pre-election		
Accurate vote count	0.16***	0.12***
Electoral status		
Loser (R)		
Independent	-0.05	0.06
Winner	0.22***	0.24***
Unexpected outcome?		
Yes	-0.53***	-0.46***
2-way interaction		
Independent × Unexpected	0.14	0.17+
Winner × Unexpected	0.21**	0.20**
Suspicious thinking		
Conspiracy beliefs		-0.50***
Political cynicism		0.08
Social mistrust		-0.06***
Information		
Political knowledge		0.33***
Constant	2.64***	2.63***
Sociodemographics	No	Yes
Region fixed effects	No	Yes
Observations	7,291	6,534
R^2	0.26	0.32

Note. Ordinary Least Squares regressions. Significance levels: + $p < 0.10$; * $p < 0.05$; ** $p < 0.01$; *** $p < 0.001$. FAIR = Perceived electoral integrity. SWD = Satisfaction with democracy. R = Reference category.

C.2 Reported Vote

Table C.2.1. Perceived Electoral Integrity and Satisfaction with Democracy, 1996 U.S. Presidential Election

	Winner-Loser Gap		Expectations	
	**Model 1.1** FAIR	**Model 2.1** SWD	**Model 1.2** FAIR	**Model 2.2** SWD
Electoral status				
Loser (R)				
Non-voter	-0.11	-0.12*	-0.11	-0.15*
Winner	0.36***	0.05	0.34***	0.05
Unexpected outcome?				
Yes			-0.08	0.00
2-way interaction				
Non-voter × Unexpected			-0.09	0.19
Winner × Unexpected			0.04	0.09
Economic evaluations				
Egotropic		0.25*		0.25*
Sociotropic		0.58***		0.58***
Information				
Political knowledge	0.32*	0.38***	0.31*	0.39***
Constant	2.62***	2.08***	2.66***	2.06***
Sociodemographics	Yes	Yes	Yes	Yes
Region fixed effects	Yes	Yes	Yes	Yes
Observations	1,367	1,329	1,363	1,325
R^2	0.08	0.12	0.08	0.12

Note. Ordinary Least Squares regressions. Significance levels: + $p < 0.10$; * $p < 0.05$; ** $p < 0.01$; *** $p < 0.001$. FAIR = Perceived electoral integrity. SWD = Satisfaction with democracy. R = Reference category.

Table C.2.2. Perceived Electoral Integrity and Satisfaction with Democracy, 2000 U.S. Presidential Election

	Winner-Loser Gap		Expectations	
	**Model 1.1** FAIR	**Model 2.1** SWD	**Model 1.2** FAIR	**Model 2.2** SWD
Electoral status				
Loser (R)				
Non-voter	-0.19	-0.08	-0.45*	-0.12
Winner	0.52***	0.10	0.30	0.12
Unexpected outcome?				
Yes			-0.23	0.04
2-way interaction				
Non-voter × Unexpected			0.40	0.06
Winner × Unexpected			0.43	-0.03
Economic evaluations				
Egotropic		-0.20		-0.20
Sociotropic		0.82**		0.82**
Information				
Political knowledge	0.06	0.60*	0.06	0.59*
Constant	1.08***	2.00***	1.26***	1.98***
Sociodemographics	Yes	Yes	Yes	Yes
Region fixed effects	Yes	Yes	Yes	Yes
Observations	621	295	621	295
R^2	0.15	0.15	0.16	0.15

Note. Ordinary Least Squares regressions. Significance levels: + $p < 0.10$; * $p < 0.05$; ** $p < 0.01$; *** $p < 0.001$. FAIR = Perceived electoral integrity. SWD = Satisfaction with democracy. R = Reference category.

Table C.2.3. Perceived Electoral Integrity and Satisfaction with Democracy, 2004 U.S. Presidential Election

	Winner-Loser Gap		Expectations	
	**Model 1.1** FAIR	**Model 2.1** SWD	**Model 1.2** FAIR	**Model 2.2** SWD
Electoral status				
Loser (R)				
Non-voter	0.38***	0.32***	0.42**	0.16+
Winner	0.90***	0.46***	0.79***	0.31***
Unexpected outcome?				
Yes			-0.27*	-0.26**
2-way interaction				
Non-voter × Unexpected			-0.22	0.31*
Winner × Unexpected			0.08	0.44**
Economic evaluations				
Egotropic		0.20		0.19
Sociotropic		0.69***		0.70***
Information				
Political knowledge	0.18	0.26*	0.15	0.26*
Constant	1.81***	1.83***	2.07***	2.00***
Sociodemographics	Yes	Yes	Yes	Yes
Region fixed effects	Yes	Yes	Yes	Yes
Observations	924	857	923	856
R^2	0.22	0.22	0.24	0.23

Note. Ordinary Least Squares regressions. Significance levels: + $p < 0.10$; * $p < 0.05$; ** $p < 0.01$; *** $p < 0.001$. FAIR = Perceived electoral integrity. SWD = Satisfaction with democracy. R = Reference category.

Table C.2.4. Perceived Electoral Integrity and Satisfaction with Democracy, 2012 U.S. Presidential Election

	Winner-Loser Gap		Expectations	
	**Model 1.1** FAIR	**Model 2.1** SWD	**Model 1.2** FAIR	**Model 2.2** SWD
Electoral status				
Loser (R)				
Non-voter	-0.05	0.07	-0.14^{+}	0.31^{***}
Winner	0.11^{**}	0.07^{+}	0.02	0.22^{***}
Unexpected outcome?				
Yes			-0.15^{**}	0.20^{***}
2-way interaction				
Non-voter × Unexpected			0.11	-0.45^{***}
Winner × Unexpected			0.08	-0.24^{*}
Suspicious thinking				
Conspiracy beliefs	-1.05^{***}	-0.43^{***}	-1.00^{***}	-0.45^{***}
Political cynicism	-0.70^{***}	-1.11^{***}	-0.71^{***}	-1.09^{***}
Social mistrust	-0.05^{**}	-0.05^{*}	-0.05^{**}	-0.05^{*}
Economic evaluations				
Egotropic		0.21^{*}		0.21^{*}
Sociotropic		0.27^{*}		0.25^{*}
Information				
Political knowledge	-0.04	-0.10	-0.03	-0.11^{+}
Constant	3.52^{***}	3.37^{***}	3.61^{***}	3.24^{***}
Sociodemographics	Yes	Yes	Yes	Yes
Region fixed effects	Yes	Yes	Yes	Yes
Observations	4,099	3,999	4,090	3,992
R^2	0.19	0.16	0.19	0.17

Note. Ordinary Least Squares regressions. Significance levels: + $p < 0.10$; * $p < 0.05$; ** $p < 0.01$; *** $p < 0.001$. FAIR = Perceived electoral integrity. SWD = Satisfaction with democracy. R = Reference category.

Table C.2.5. Perceived Electoral Integrity and Satisfaction with Democracy, 2016 U.S. Presidential Election

	Winner-Loser Gap		Expectations	
	**Model 1.1**<br>FAIR	**Model 2.1**<br>SWD	**Model 1.2**<br>FAIR	**Model 2.2**<br>SWD
Electoral status				
Loser (R)				
Non-voter	-0.09*	0.05	0.10	0.06
Winner	0.09*	0.31***	0.08	0.25***
Unexpected outcome?				
Yes			-0.02	-0.09
2-way interaction				
Non-voter × Unexpected			-0.28**	-0.01
Winner × Unexpected			0.06	0.09
Suspicious thinking				
Conspiracy beliefs	-0.15*	0.02	-0.19**	-0.00
Political cynicism	-0.56***	-0.62***	-0.60***	-0.65***
Social mistrust	-0.08***	-0.02	-0.08***	-0.02
Economic evaluations				
Egotropic		-0.07		-0.07
Sociotropic		0.53***		0.55***
Information				
Political knowledge	0.19**	0.08	0.21**	0.09
Constant	3.10***	2.54***	3.14***	2.62***
Sociodemographics	Yes	Yes	Yes	Yes
Region fixed effects	Yes	Yes	Yes	Yes
Observations	3,101	3,043	3,096	3,038
R^2	0.13	0.10	0.14	0.10

Note. Ordinary Least Squares regressions. Significance levels: + $p < 0.10$; * $p < 0.05$; ** $p < 0.01$; *** $p < 0.001$. FAIR = Perceived electoral integrity. SWD = Satisfaction with democracy. R = Reference category.

Table C.2.6. Perceived Electoral Integrity and Satisfaction with Democracy, 2020 U.S. Presidential Election

	Winner-Loser Gap		Expectations	
	**Model 1.1** FAIR	**Model 2.1** SWD	**Model 1.2** FAIR	**Model 2.2** SWD
Electoral status				
Loser (R)				
Non-voter	0.29***	-0.03	0.33***	0.07
Winner	0.71***	-0.05	0.46***	-0.03
Unexpected outcome?				
Yes			-0.34***	0.00
2-way interaction				
Non-voter × Unexpected			-0.24*	-0.17*
Winner × Unexpected			0.24**	-0.01
Suspicious thinking				
Conspiracy beliefs	-0.62***	-0.47***	-0.55***	-0.46***
Political cynicism	-0.05	-0.51***	-0.08	-0.50***
Social mistrust	-0.06**	-0.01	-0.06**	-0.01
Economic evaluations				
Egotropic		0.07		0.07
Sociotropic		0.30***		0.32***
Information				
Political knowledge	0.37***	-0.10	0.32***	-0.12
Constant	2.68***	2.87***	2.93***	2.83***
Sociodemographics	Yes	Yes	Yes	Yes
Region fixed effects	Yes	Yes	Yes	Yes
Observations	6,535	6,464	6,510	6,443
R^2	0.28	0.08	0.32	0.08

Note. Ordinary Least Squares regressions. Significance levels: + $p < 0.10$; * $p < 0.05$; ** $p < 0.01$; *** $p < 0.001$. FAIR = Perceived electoral integrity. SWD = Satisfaction with democracy. R = Reference category.

Table C.2.7. Perceived Electoral Integrity (Pre/Post), 2020 U.S. Presidential Election

	**Model 1**<br>No controls	**Model 2**<br>Controls
Pre-election		
Accurate vote count	0.15***	0.11***
Electoral status		
Loser (R)		
Non-voter	0.19**	0.31***
Winner	0.47***	0.42***
Unexpected outcome?		
Yes	-0.32***	-0.30***
2-way interaction		
Non-voter × Unexpected	-0.23**	-0.24*
Winner × Unexpected	0.23**	0.24**
Suspicious thinking		
Conspiracy beliefs		-0.47***
Political cynicism		0.07
Social mistrust		-0.05**
Information		
Political knowledge		0.30***
Constant	2.46***	2.50***
Sociodemographics	No	Yes
Region fixed effects	No	Yes
Observations	7,238	6,501
R^2	0.29	0.34

Note. Ordinary Least Squares regressions. Significance levels: + $p < 0.10$; * $p < 0.05$; ** $p < 0.01$; *** $p < 0.001$. FAIR = Perceived electoral integrity. SWD = Satisfaction with democracy. R = Reference category.

D Regression Tables: 3-Way Interaction

One could argue that the influence of unexpected outcomes on perceived electoral integrity and satisfaction with democracy will be affected by the tendency to harbour suspicions about other people's motives and actions. More precisely, the gap between unexpected and expected losers should be largest for those who are most suspicious about the intents of political actors and other people more broadly. For this reason, a set of models for the 2012, 2016, and 2020 elections, including a three-way interaction between respondents' electoral status, outcome unexpectedness, and a suspicion index summarizing respondents' answers on the conspiratorial thinking, political cynicism, and social mistrust items, were estimated. Regression outputs for these three models are presented in tables D.1.1 to D.1.3 (party identification) and D.2.1 to D.2.3 (reported vote).

The *Electoral status* × *Unexpectedness* × *Suspicion* interaction is only statistically significant in the case of the 2020 presidential election for perceived electoral integrity: as the level of suspicious thinking increases among losers, the size of the gap between unexpected and expected losers increases as well. While there is no gap between unexpected and expected losers among the least suspicious respondents, the gap between unexpected and expected losers among the most suspicious respondents is substantial (-0.87 points). This can be seen in Figure D.1. However, when we look separately at respondents according to their perceptions of election closeness, we see that the interaction effect remains significant only among those who predicted an easy victory for their candidate (-0.68 points), i.e., those that were the most surprised/disappointed by the outcome.

Figure D.1. Unexpected-Expected Gap in Perceived Electoral Integrity According to Respondent's Electoral Status and Level of Suspicious Thinking, 2012–2020

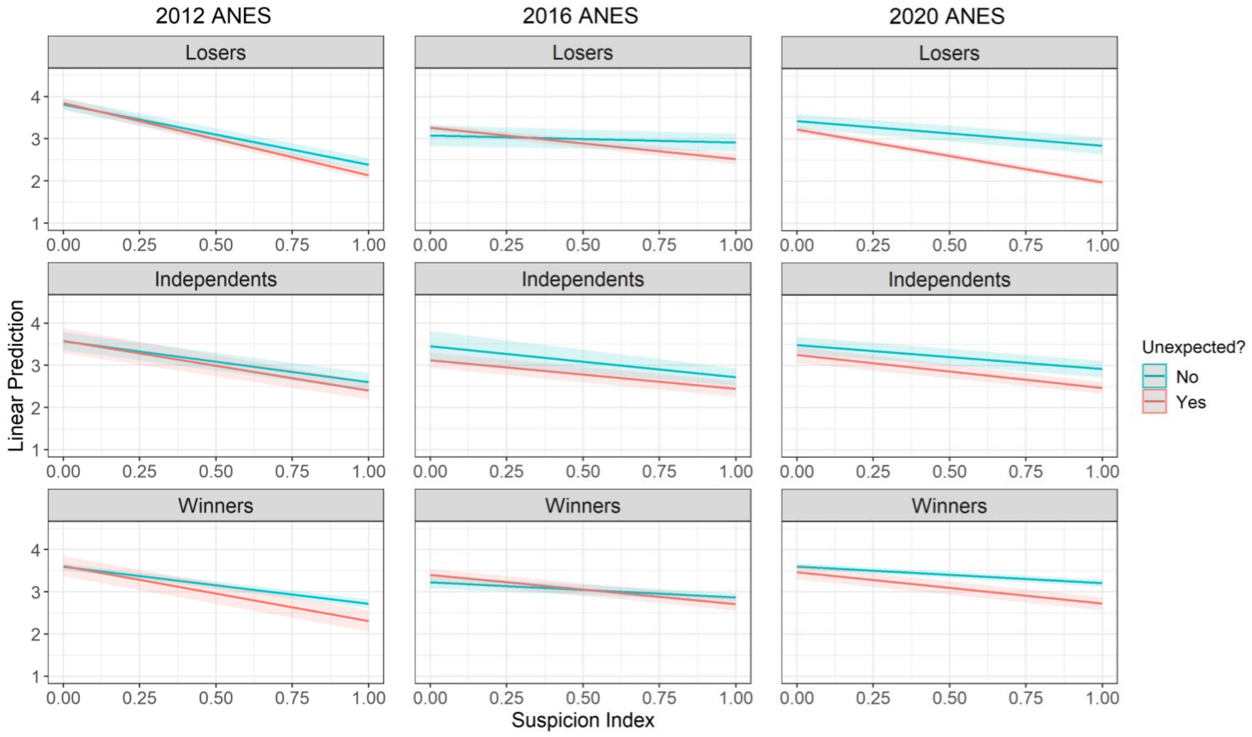

Note. Estimations based on results from tables D.1.1 to D.1.3. The shaded areas represent the 95 per cent confidence intervals.

D.1 Party Identification

Table D.1.1. Perceived Electoral Integrity and Satisfaction with Democracy, 2012 U.S. Presidential Election

	**Model 1** FAIR	**Model 2** SWD
Electoral status		
Loser (R)		
Independent	-0.25	-0.55***
Winner	-0.23+	-0.32**
Unexpected outcome?		
Yes	0.03	-0.09
2-way interactions		
Independent × Unexpected	-0.01	0.29
Winner × Unexpected	-0.00	0.14
Independent × Suspicion	0.47	0.99***
Winner × Suspicion	0.57*	0.74***
Unexpected × Suspicion	-0.27	0.30
3-way interaction		
Independent × Unexpected × Suspicion	0.04	-0.93*
Winner × Unexpected × Suspicion	-0.17	-0.54
Suspicious thinking		
Suspicion index	-1.44***	-1.52***
Economic evaluations		
Egotropic		0.17*
Sociotropic		0.38***
Information		
Political knowledge	0.02	-0.15**
Constant	3.22***	3.25***
Sociodemographics	Yes	Yes
Region fixed effects	Yes	Yes
Observations	4,424	4,314
R^2	0.20	0.17

Note. Ordinary Least Squares regressions. Significance levels: + $p < 0.10$; * $p < 0.05$; ** $p < 0.01$; *** $p < 0.001$. FAIR = Perceived electoral integrity. SWD = Satisfaction with democracy. R = Reference category.

Table D.1.2. Perceived Electoral Integrity and Satisfaction with Democracy, 2016 U.S. Presidential Election

	**Model 1**<br>FAIR	**Model 2**<br>SWD
Electoral status		
Loser (R)		
Independent	0.36	0.79^{**}
Winner	0.16	0.58^{**}
Unexpected outcome?		
Yes	0.19	0.08
2-way interactions		
Independent × Unexpected	-0.50	-0.51^{+}
Winner × Unexpected	-0.01	-0.22
Independent × Suspicion	-0.54	-1.40^{***}
Winner × Suspicion	-0.21	-0.67^{*}
Unexpected × Suspicion	-0.58^{*}	-0.28
3-way interaction		
Independent × Unexpected × Suspicion	0.60	0.73
Winner × Unexpected × Suspicion	0.25	0.44
Suspicious thinking		
Suspicion index	-0.17	0.24
Economic evaluations		
Egotropic		-0.05
Sociotropic		0.57^{***}
Information		
Political knowledge	0.19^{**}	0.06
Constant	2.48^{***}	2.04^{***}
Sociodemographics	Yes	Yes
Region fixed effects	Yes	Yes
Observations	3,098	3,042
R^2	0.13	0.10

Note. Ordinary Least Squares regressions. Significance levels: + $p < 0.10$; * $p < 0.05$; ** $p < 0.01$; *** $p < 0.001$. FAIR = Perceived electoral integrity. SWD = Satisfaction with democracy. R = Reference category.

Table D.1.3. Perceived Electoral Integrity and Satisfaction with Democracy, 2020 U.S. Presidential Election

	**Model 1** FAIR	**Model 2** SWD
Electoral status		
Loser (R)		
Independent	0.06	0.25
Winner	0.18^{+}	0.03
Unexpected outcome?		
Yes	-0.19	0.21
2-way interactions		
Independent × Unexpected	-0.03	-0.30
Winner × Unexpected	0.06	-0.21
Independent × Suspicion	0.01	-0.87^{*}
Winner × Suspicion	0.19	-0.27
Unexpected × Suspicion	-0.67^{*}	-0.63^{+}
3-way interaction		
Independent × Unexpected × Suspicion	0.45	0.74
Winner × Unexpected × Suspicion	0.32	0.45
Suspicious thinking		
Suspicion index	-0.58^{*}	-0.34
Economic evaluations		
Egotropic		0.04
Sociotropic		0.30^{***}
Information		
Political knowledge	0.42^{***}	-0.14^{+}
Constant	2.78^{***}	2.62^{***}
Sociodemographics	Yes	Yes
Region fixed effects	Yes	Yes
Observations	6,543	6,474
R^2	0.30	0.08

Note. Ordinary Least Squares regressions. Significance levels: + $p < 0.10$; * $p < 0.05$; ** $p < 0.01$; *** $p < 0.001$. FAIR = Perceived electoral integrity. SWD = Satisfaction with democracy. R = Reference category.

Table D.1.4. Perceived Electoral Integrity (Pre/Post), 2020 U.S. Presidential Election

	**Model 1**<br>No controls	**Model 2**<br>Controls
Pre-election		
Accurate vote count	0.12^{***}	0.11^{***}
Electoral status		
Loser (R)		
Independent	-0.13	0.04
Winner	0.17^{+}	0.17^{+}
Unexpected outcome?		
Yes	-0.30^{**}	-0.16
2-way interactions		
Independent × Unexpected	-0.03	-0.04
Winner × Unexpected	0.02	0.01
Independent × Suspicion	0.21	0.05
Winner × Suspicion	0.14	0.16
Unexpected × Suspicion	-0.38	-0.65^{**}
3-way interaction		
Independent × Unexpected × Suspicion	0.32	0.44
Winner × Unexpected × Suspicion	0.34	0.40
Suspicious thinking		
Suspicion index	-0.67^{**}	-0.40^{+}
Information		
Political knowledge		0.40^{***}
Constant	3.09^{***}	2.40^{***}
Sociodemographics	No	Yes
Region fixed effects	No	Yes
Observations	7,131	6,534
R^2	0.29	0.32

Note. Ordinary Least Squares regressions. Significance levels: + $p < 0.10$; * $p < 0.05$; ** $p < 0.01$; *** $p < 0.001$. FAIR = Perceived electoral integrity. SWD = Satisfaction with democracy. R = Reference category.

D.2 Reported Vote

Table D.2.1. Perceived Electoral Integrity and Satisfaction with Democracy, 2012 U.S. Presidential Election

	**Model 1** FAIR	**Model 2** SWD
Electoral status		
Loser (R)		
Non-voter	-0.46*	0.30^{+}
Winner	-0.30**	0.07
Unexpected outcome?		
Yes	-0.02	0.29^{+}
2-way interactions		
Non-voter × Unexpected	-0.31	-0.38
Winner × Unexpected	0.06	-0.03
Non-voter × Suspicion	0.67^{+}	0.05
Winner × Suspicion	0.76**	0.36
Unexpected × Suspicion	-0.22	-0.11
3-way interaction		
Non-voter × Unexpected × Suspicion	0.61	-0.12
Winner × Unexpected × Suspicion	-0.09	-0.55
Suspicious thinking		
Suspicion index	-1.56***	-1.10***
Economic evaluations		
Egotropic		0.21*
Sociotropic		0.28*
Information		
Political knowledge	-0.01	-0.15*
Constant	3.39***	2.86***
Sociodemographics	Yes	Yes
Region fixed effects	Yes	Yes
Observations	4,090	3,992
R^2	0.20	0.17

Note. Ordinary Least Squares regressions. Significance levels: + $p < 0.10$; * $p < 0.05$; ** $p < 0.01$; *** $p < 0.001$. FAIR = Perceived electoral integrity. SWD = Satisfaction with democracy. R = Reference category.

Table D.2.2. Perceived Electoral Integrity and Satisfaction with Democracy, 2016 U.S. Presidential Election

	**Model 1** FAIR	**Model 2** SWD
Electoral status		
Loser (R)		
Non-voter	0.08	0.17
Winner	0.05	0.37*
Unexpected outcome?		
Yes	0.14	-0.16
2-way interactions		
Non-voter × Unexpected	-0.29	-0.02
Winner × Unexpected	-0.20	0.04
Non-voter × Suspicion	0.04	-0.11
Winner × Suspicion	0.08	-0.12
Unexpected × Suspicion	-0.42	0.24
3-way interaction		
Non-voter × Unexpected × Suspicion	0.11	-0.15
Winner × Unexpected × Suspicion	0.61+	-0.07
Suspicious thinking		
Suspicion index	-0.49*	-0.38
Economic evaluations		
Egotropic		-0.04
Sociotropic		0.63***
Information		
Political knowledge	0.17*	0.04
Constant	2.67***	2.28***
Sociodemographics	Yes	Yes
Region fixed effects	Yes	Yes
Observations	3,096	3,038
R^2	0.14	0.09

Note. Ordinary Least Squares regressions. Significance levels: + $p < 0.10$; * $p < 0.05$; ** $p < 0.01$; *** $p < 0.001$. FAIR = Perceived electoral integrity. SWD = Satisfaction with democracy. R = Reference category.

Table D.2.3. Perceived Electoral Integrity and Satisfaction with Democracy, 2020 U.S. Presidential Election

	**Model 1**<br>FAIR	**Model 2**<br>SWD
Electoral status		
Loser (R)		
Independent	0.26	-0.17
Winner	0.39^{*}	-0.38^{**}
Non-voter outcome?		
Yes	-0.02	-0.19
2-way interactions		
Independent × Non-voter	-0.51^{+}	0.06
Winner × Non-voter	0.03	0.48^{*}
Independent × Suspicion	0.12	0.50
Winner × Suspicion	0.17	0.70^{*}
Non-voter × Suspicion	-0.67^{+}	0.37
3-way interaction		
Independent × Non-voter × Suspicion	0.54	-0.45
Winner × Non-voter × Suspicion	0.41	-1.00^{**}
Suspicious thinking		
Suspicion index	-0.59^{+}	-1.29^{***}
Economic evaluations		
Egotropic		0.05
Sociotropic		0.33^{***}
Information		
Political knowledge	0.37^{***}	-0.12
Constant	2.69^{***}	2.94^{***}
Sociodemographics	Yes	Yes
Region fixed effects	Yes	Yes
Observations	6,510	6,443
R^2	0.32	0.08

Note. Ordinary Least Squares regressions. Significance levels: + $p < 0.10$; * $p < 0.05$; ** $p < 0.01$; *** $p < 0.001$. FAIR = Perceived electoral integrity. SWD = Satisfaction with democracy. R = Reference category.

Table D.2.4. Perceived Electoral Integrity (Pre/Post), 2020 U.S. Presidential Election

	**Model 1**<br>No controls	**Model 2**<br>Controls
Pre-election		
Accurate vote count	0.11^{***}	0.11^{***}
Electoral status		
Loser (R)		
Non-voter	0.06	0.24
Winner	0.35^{*}	0.33^{*}
Unexpected outcome?		
Yes	-0.13	-0.03
2-way interactions		
Non-voter × Unexpected	-0.39	-0.50^{+}
Winner × Unexpected	0.04	0.05
Non-voter × Suspicion	0.27	0.13
Winner × Suspicion	0.18	0.23
Unexpected × Suspicion	-0.38	-0.58^{+}
3-way interaction		
Non-voter × Unexpected × Suspicion	0.34	0.52
Winner × Unexpected × Suspicion	0.39	0.39
Suspicious thinking		
Suspicion index	-0.67^{*}	-0.48
Information		
Political knowledge		0.35^{***}
Constant	2.93^{***}	2.36^{***}
Sociodemographics	No	Yes
Region fixed effects	No	Yes
Observations	7,083	6,501
R^2	0.31	0.34

Note. Ordinary Least Squares regressions. Significance levels: + $p < 0.10$; * $p < 0.05$; ** $p < 0.01$; *** $p < 0.001$. FAIR = Perceived electoral integrity. SWD = Satisfaction with democracy. R = Reference category.

E Conservative vs Liberal Conspiracy Beliefs

Specific conspiracy beliefs might not have the same effect on conservatives/Republicans and liberals/Democrats.[6] Following Miller, Saunders and Farhart (2016), the 2012 and 2016 conspiracy items were divided between those expected to be more attractive to conservatives/Republicans (i.e., Obama's birthplace and death panels in 2012; Obama's religion in 2016) and those expected to be more attractive to liberals/Democrats (i.e., 9/11 knowledge and intentional flooding in 2012; 9/11 knowledge in 2016). The variables for conspiracy beliefs were standardized (i.e., mean = 0, sd = 1) to ease comparison. Generally speaking, both "conservative" and "liberal" conspiracy beliefs have a negative impact on perceived electoral integrity and satisfaction with democracy. Interestingly, conspiracy beliefs that are potentially more attractive to conservative/Republican respondents seem to have a larger negative influence on perceptions of electoral integrity than conspiracy beliefs that are potentially more attractive to liberal/Democratic respondents in 2012 (Republican defeat) and a smaller negative influence in 2016 (Republican victory). We can also see that respondents holding conservative conspiracy beliefs were *more* satisfied with democracy in 2016, all else being equal, while respondents holding liberal conspiracy beliefs were less satisfied with democracy.

[6] Obviously, partisan and ideological motivations play a role in the endorsement of conspiracy theories. That being said, conspiratorial ideation are not necessarily extinguished by partisan loyalty when in-group members are purportedly implicated in secretive and malevolent plots (Enders and Smallpage, 2019).

E.1 Party Identification

Table E.1.1. Perceived Electoral Integrity and Satisfaction with Democracy, 2012 U.S. Presidential Election

	FAIR		SWD	
	**Model 1.1**<br>1 item	**Model 1.2**<br>2 items	**Model 2.2**<br>1 item	**Model 2.2**<br>2 items
Electoral status				
Loser (R)				
Independent	-0.08	-0.10^{+}	-0.16**	-0.14**
Winner	-0.01	-0.03	0.00	0.03
Unexpected outcome?				
Yes	-0.17***	-0.15***	0.01	-0.02
2-way interaction				
Independent × Unexpected	0.07	0.06	-0.04	-0.03
Winner × Unexpected	0.09	0.08	-0.08	-0.06
Suspicion				
Conspiracy beliefs	-0.20***		-0.10***	
Conspiracy beliefs (Con)		-0.17***		-0.02^{+}
Conspiracy beliefs (Lib)		-0.10***		-0.10***
Political cynicism	-0.70***	-0.71***	-1.02***	-1.00***
Social mistrust	-0.06***	-0.06***	-0.02	-0.02
Economic evaluations				
Sociotropic			0.34***	0.40***
Egotropic			0.18**	0.18**
Information				
Political knowledge	-0.03	-0.03	-0.17***	-0.17***
Constant	3.33***	3.34***	3.30***	3.25***
Sociodemographics	Yes	Yes	Yes	Yes
Region fixed effects	Yes	Yes	Yes	Yes
Observations	4,424	4,424	4,314	4,314
R^2	0.18	0.18	0.18	0.18

Note. Ordinary Least Squares regressions. Significance levels: + $p < 0.10$; * $p < 0.05$; ** $p < 0.01$; *** $p < 0.001$. Measures for conspiracy beliefs were standardized. FAIR = Perceived electoral integrity. SWD = Satisfaction with democracy. R = Reference category.

Table E.1.2. Perceived Electoral Integrity and Satisfaction with Democracy, 2016 U.S. Presidential Election

	FAIR		SWD	
	**Model 1.1** 1 item	**Model 1.2** 2 items	**Model 2.2** 1 item	**Model 2.2** 2 items
Electoral status				
Loser (R)				
Independent	0.05	0.04	-0.03	-0.03
Winner	0.03	0.00	0.27***	0.23***
Unexpected outcome?				
Yes	-0.10*	-0.10+	-0.08	-0.07
2-way interaction				
Independent × Unexpected	-0.18*	-0.18*	0.05	0.05
Winner × Unexpected	0.12+	0.12*	0.06	0.07
Suspicion				
Conspiracy beliefs	-0.07***		0.01	
Conspiracy beliefs (Con)		-0.04**		0.05**
Conspiracy beliefs (Lib)		-0.07***		-0.08***
Political cynicism	-0.58***	-0.55***	-0.61***	-0.55***
Social mistrust	-0.07***	-0.07***	-0.02	-0.02
Economic evaluations				
Sociotropic			0.53***	0.55***
Egotropic			-0.04	-0.04
Information				
Political knowledge	0.20***	0.20***	0.11+	0.10+
Constant	3.04***	3.07***	2.61***	2.63***
Sociodemographics	Yes	Yes	Yes	Yes
Region fixed effects	Yes	Yes	Yes	Yes
Observations	3,098	3,098	3,042	3,042
R^2	0.13	0.14	0.11	0.12

Note. Ordinary Least Squares regressions. Significance levels: + $p < 0.10$; * $p < 0.05$; ** $p < 0.01$; *** $p < 0.001$. Measures for conspiracy beliefs were standardized. FAIR = Perceived electoral integrity. SWD = Satisfaction with democracy. R = Reference category.

E.2 Reported Vote

Table E.2.1. Perceived Electoral Integrity and Satisfaction with Democracy, 2012 U.S. Presidential Election

	FAIR		SWD	
	**Model 1.1** 1 item	**Model 1.2** 2 items	**Model 2.2** 1 item	**Model 2.2** 2 items
Electoral status				
Loser (R)				
Non-voter	-0.10^{+}	-0.11*	0.17**	0.18***
Winner	0.02	-0.00	0.15***	0.19***
Unexpected outcome?				
Yes	-0.18***	-0.17***	0.15***	0.12**
2-way interaction				
Non-voter × Unexpected	0.15^{+}	0.15^{+}	-0.34***	-0.33***
Winner × Unexpected	0.19*	0.17*	-0.23**	-0.19*
Suspicion				
Conspiracy beliefs	-0.19***		-0.10***	
Conspiracy beliefs (Con)		-0.16***		-0.02
Conspiracy beliefs (Lib)		-0.10***		-0.11***
Political cynicism	-0.70***	-0.71***	-1.03***	-1.01***
Social mistrust	-0.07***	-0.07***	-0.02	-0.02
Economic evaluations				
Sociotropic			0.29***	0.34***
Egotropic			0.21***	0.21***
Information				
Political knowledge	-0.03	-0.03	-0.17***	-0.17***
Constant	3.38***	3.39***	3.15***	3.09***
Sociodemographics	Yes	Yes	Yes	Yes
Region fixed effects	Yes	Yes	Yes	Yes
Observations	4,090	4,090	3,992	3,992
R^2	0.19	0.19	0.18	0.18

Note. Ordinary Least Squares regressions. Significance levels: + $p < 0.10$; * $p < 0.05$; ** $p < 0.01$; *** $p < 0.001$. Measures for conspiracy beliefs were standardized. FAIR = Perceived electoral integrity. SWD = Satisfaction with democracy. R = Reference category.

Table E.2.2. Perceived Electoral Integrity and Satisfaction with Democracy, 2016 U.S. Presidential Election

	FAIR		SWD	
	**Model 1.1** 1 item	**Model 1.2** 2 items	**Model 2.2** 1 item	**Model 2.2** 2 items
Electoral status				
Loser (R)				
Non-voter	0.07	0.06	-0.00	-0.03
Winner	0.06	0.04	0.26***	0.22***
Unexpected outcome?				
Yes	-0.05	-0.04	-0.14**	-0.13**
2-way interaction				
Non-voter × Unexpected	-0.22***	-0.22**	0.10	0.11
Winner × Unexpected	0.07	0.07	0.11+	0.12+
Suspicion				
Conspiracy beliefs	-0.07***		-0.00	
Conspiracy beliefs (Con)		-0.04**		0.04*
Conspiracy beliefs (Lib)		-0.07***		-0.08***
Political cynicism	-0.58***	-0.55***	-0.62***	-0.57***
Social mistrust	-0.06***	-0.06***	-0.02	-0.02
Economic evaluations				
Sociotropic			0.56***	0.58***
Egotropic			-0.01	-0.01
Information				
Political knowledge	0.17***	0.17**	0.09	0.08
Constant	3.04***	3.07***	2.62***	2.66***
Sociodemographics	Yes	Yes	Yes	Yes
Region fixed effects	Yes	Yes	Yes	Yes
Observations	3,096	3,096	3,038	3,038
R^2	0.13	0.14	0.11	0.12

Note. Ordinary Least Squares regressions. Significance levels: + $p < 0.10$; * $p < 0.05$; ** $p < 0.01$; *** $p < 0.001$. Measures for conspiracy beliefs were standardized. FAIR = Perceived electoral integrity. SWD = Satisfaction with democracy. R = Reference category.

F Expected Closeness

F.1 Party Identification

Figure F.1.1. Unexpected-Expected Gap in Perceived Electoral Integrity According to Electoral Status and Expected Closeness, 1996–2020

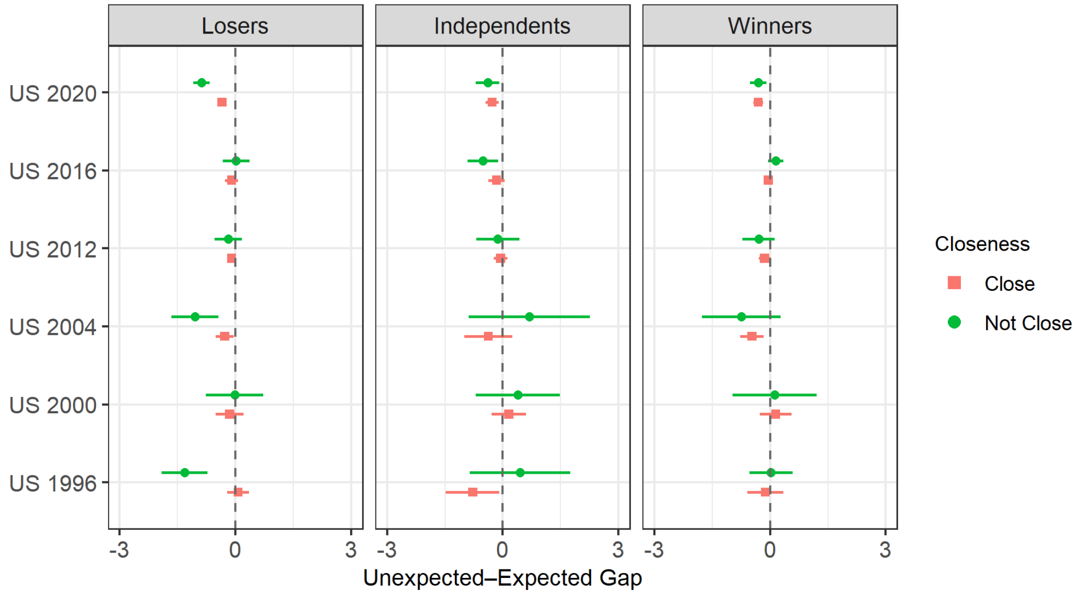

Note. The vertical lines represent the 95 per cent confidence intervals.

Figure F.1.2. Unexpected-Expected Gap in Satisfaction with Democracy According to Electoral Status and Expected Closeness, 1996–2020

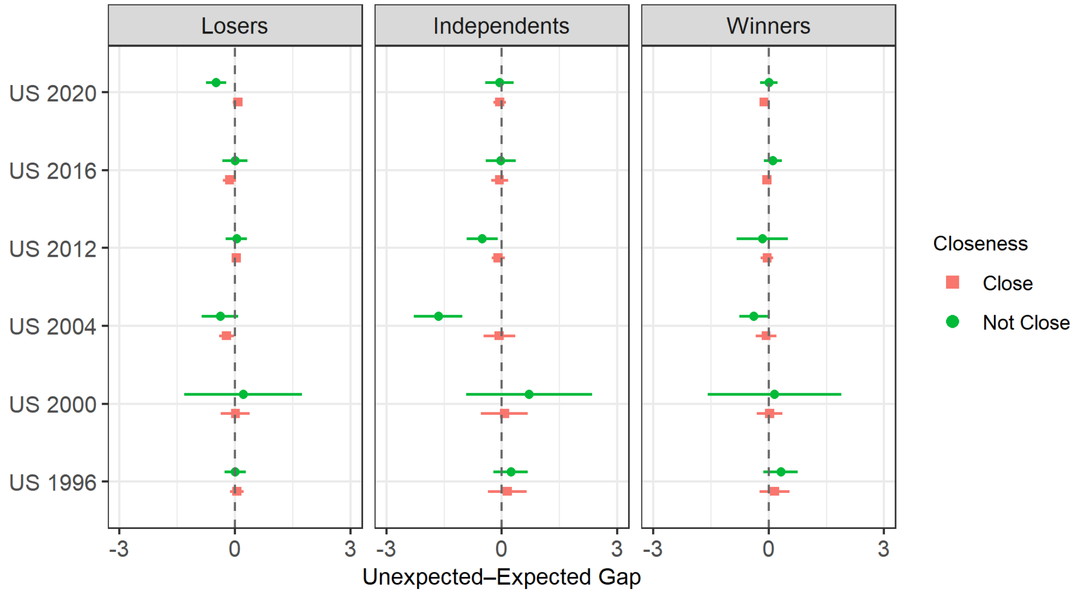

Note. The vertical lines represent the 95 per cent confidence intervals.

F.2 Reported Vote

Figure F.2.1. Unexpected-Expected Gap in Perceived Electoral Integrity According to Electoral Status and Expected Closeness, 1996–2020

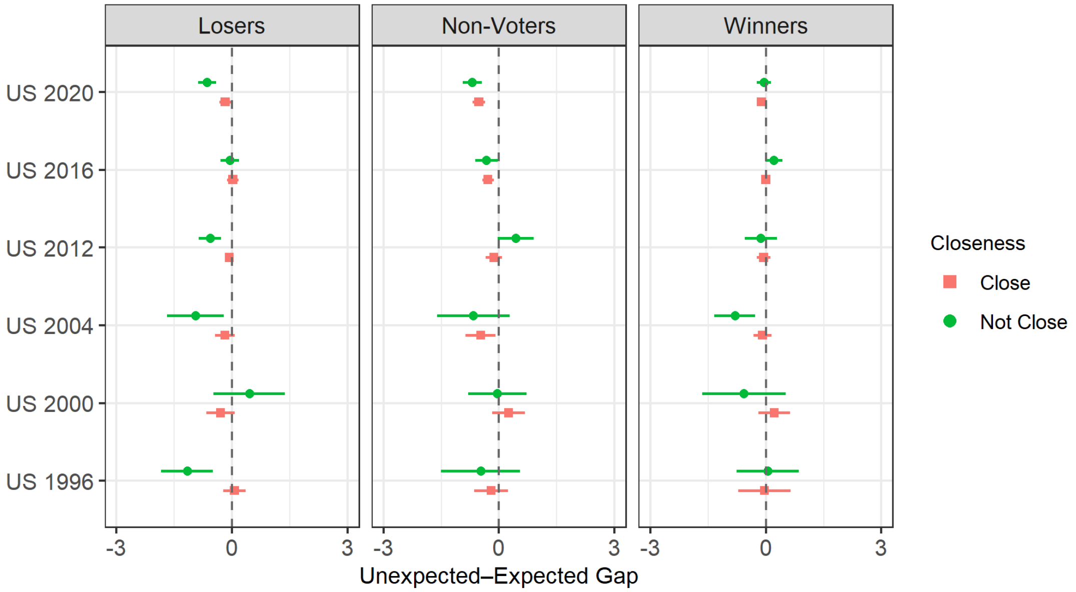

Note. The vertical lines represent the 95 per cent confidence intervals.

Figure F.2.2. Unexpected-Expected Gap in Satisfaction with Democracy According to Electoral Status and Expected Closeness, 1996–2020

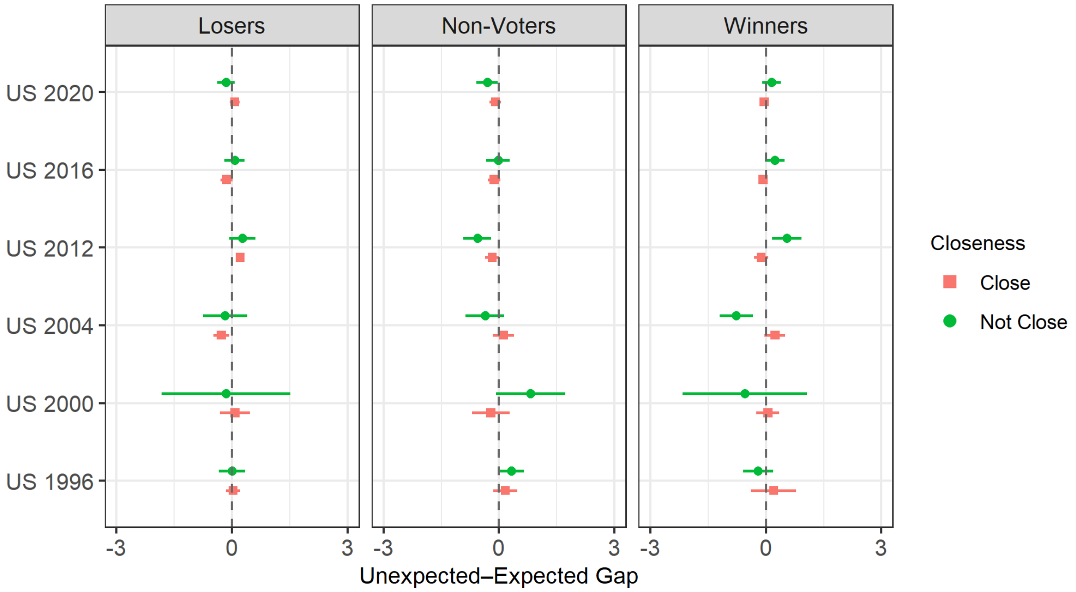

Note. The vertical lines represent the 95 per cent confidence intervals.

G Party Identification Strength

Figure G.1. Unexpected-Expected Gap in Perceived Electoral Integrity According to Electoral Status and Strength of Party Identification, 1996–2020

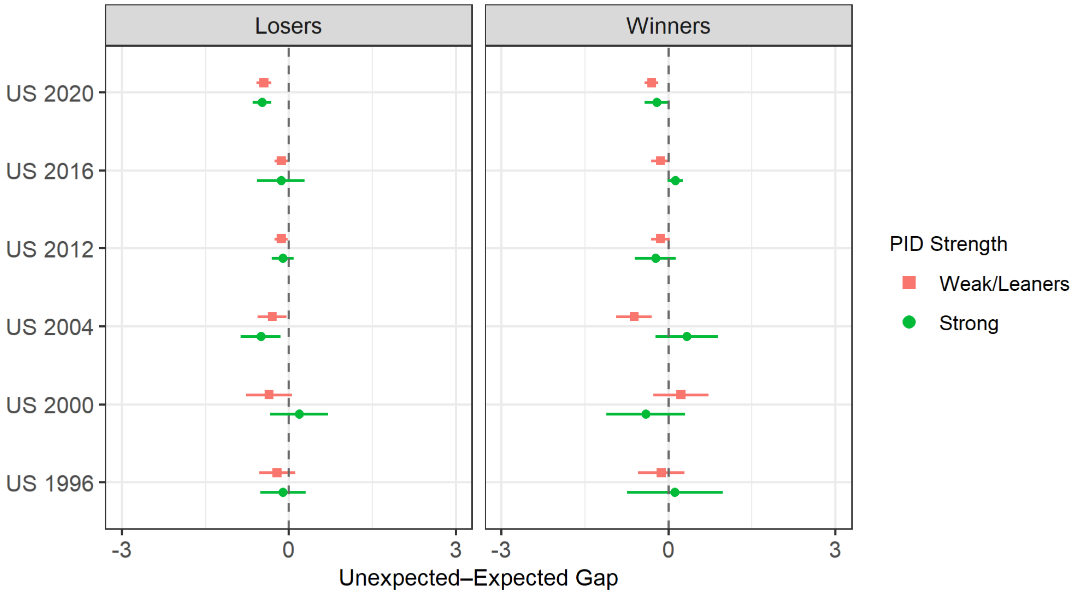

Note. The vertical lines represent the 95 per cent confidence intervals.

Figure G.2. Unexpected-Expected Gap in Satisfaction with Democracy According to Electoral Status and Strength of Party Identification, 1996–2020

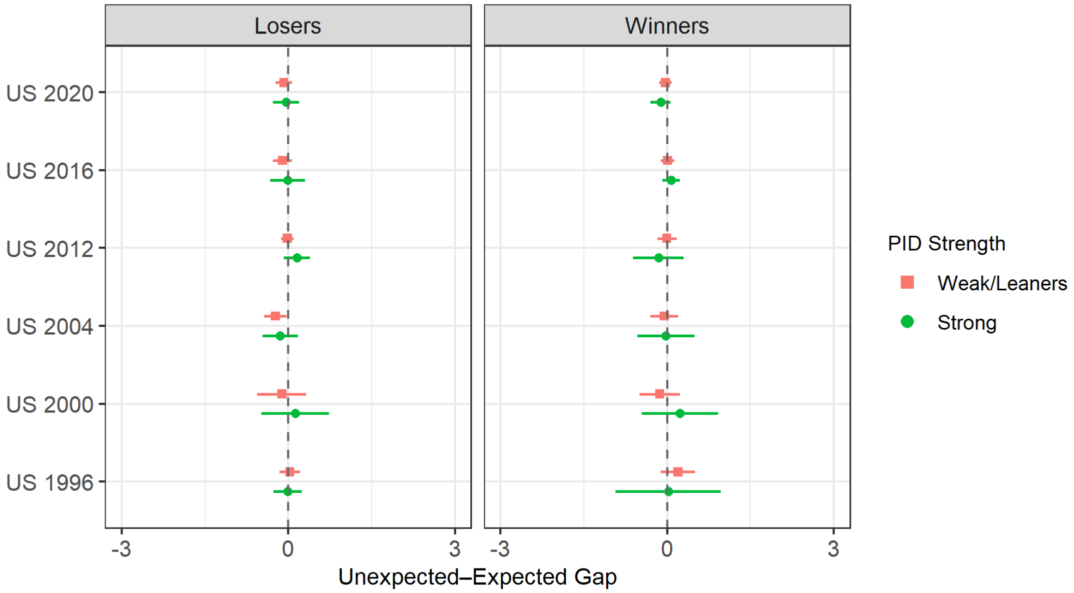

Note. The vertical lines represent the 95 per cent confidence intervals.

H The Influence of Polls

As mentioned by Irwin and van Holsteyn (2002, 92), "some researchers have argued that expectations are acquired cognitively (e.g., based on polls), while others claim that they are affective (i.e., wishful thinking)." The reality certainly lies somewhere in between those two claims. The literature on electoral expectations and political evaluations of competing parties or candidates is replete with examples of wishful thinking and motivated reasoning. At the same time, a number of studies have demonstrated that citizens *attentive* to polling information tend to adjust their forecasts in the direction of the polls, although the influence of vote intention surveys is not necessarily a strong one (Daschmann, 2000; Meffert et al., 2011). Nevertheless, evidence to that effect have been provided for multiple elections, including the 1994 Dutch parliamentary election (Irwin and van Holsteyn, 2002), the 2005 German parliamentary election (Faas, Mackenrodt and Schmitt-Beck, 2008), and the 2011 Croatian parliamentary election (Ferić and Posavec, 2013). Morwitz and Pluzinski (1996) have proposed a set of hypotheses based on cognitive consistency theories to explore the impact of polls (which are presented as a form of "reality constraint") on citizens' electoral expectations, preferences, and voting behaviour. They predict that voters in a state of dissonance prior to poll exposure (i.e., one expects his/her preferred candidate to lose) will change their preferences when polling information confirms their expectations (the preferred candidate is trailing) and that they will change their expectations when polling information indicate that their preferred candidate is ahead. When voters in a state of cognitive consistency (i.e., when preferences match expectations) encounter polls going against their beliefs, they will tend to ignore polling information and maintain their pre-existing expectations and preferences. In other words, agreeable polling information will simply reinforce or confirm pre-existing expectations and preferences. Through experiments conducted during the 1992 U.S. presidential election and the 1993 New York City mayoral election, they find that individuals appear to "use the results of political polls to reduce cognitive dissonance" (Morwitz and Pluzinski, 1996, 62). When expectations are in line with preferences, however, exposure to (confirming or disconfirming) polls do not yield any influence over voters' attitudes or intents.

To use Morwitz and Pluzinski's (1996) terminology, expected losers and unexpected winners are in a state of cognitive dissonance (i.e., there is a mismatch between preferences and expectations), while unexpected losers and expected winners are in a state of cognitive consistency (i.e., there is a match between preferences and expectations). To determine whether or not polling information is congruent with the beliefs of ANES respondents, the results of vote intention surveys between 1996 and 2020 were collected. More precisely, I use the daily poll averages computed by Jennings and Wlezien (2017) for the 1996–2016 period as well as Wikipedia's list of nationwide public opinion polls that were conducted during the 2020 election campaign (polls released on the same day were averaged).[7] Figure H.1 shows the daily averages of vote intentions for both the Democratic and Republican candidates in the last 90 days of the campaign for each election as well as the evolution of respondents' forecasts over the ANES pre-election interview period. From eyeballing the graph, we can see that voters seem to have an easier time at forecasting the outcome when an incumbent is running for a second term (1996, 2004, 2012) as indicated by the pale green line (i.e., the percentage of correct forecasts from ANES respondents), although this was not the case in 2020, where voters were apparently much more divided on the likely winner of the election.

From these data, it was determined which candidate was ahead in the "horse-race" on a given day and how big was his/her lead over the runner-up. In order to simulate a realistic window of exposure, a three-day average was computed for each ANES respondent (e.g., for a respondent interviewed on October 31st, vote intentions polls released on the 29th, 30th and 31st of October were averaged).[8] This three-day window is somewhat arbitrary, but it is worth noting that Faas, Mackenrodt and Schmitt-Beck (2008) found the effect of polls on expectations to be strongest for a two-day lag. Ansolabehere and Iyengar (1994, 421) have noted that perceptions of electoral prospects based on polls "are not sensitive to the magnitude of the spread among the candidates. Individuals seem to use a simple 'who's leading' rule rather than the size of the lead." However, we might expect even mildly sophisticated voters to interpret tight polling results as inconclusive (e.g., a one-point lead with an error margin of two or three percentage points basically reflects a toss-up race). For polling information to be considered as incongruent with respondents' expectations, the margin

[7] When the release date of a poll was not mentioned, the end date of the fieldwork was used instead.

[8] The matching of the polling data with ANES respondents was accomplished through the `nearmrg` Stata package with the `lower` option. This package performs nearest-match merging of two datasets (see Booth 2003).

between the first-place and second-place candidates had to be greater than two percentage points. Otherwise, we can hypothesized that the polls do not confirm *nor* disconfirm pre-existing expectations (i.e., these polls are easier to discount).

In 1996, about 8.2 per cent of respondents (the figure is similar if we remove the 2 pp threshold as Bill Clinton's lead never dropped below eight percentage points during the ANES interview period) were in a state of cognitive consistency and (presumably) exposed to incongruent polling information—e.g., I support the Republican Party and I think the Republican candidate is going to win, but polls indicate a relatively clear advantage (lead > 2 pp) for the Democratic candidate. This was the case of 20.7 per cent of respondents (35.6 per cent when removing the 2 pp threshold) in the 2000 election, 20.9 per cent (36.4 per cent) in the 2004 election, 1.4 per cent (49.8 per cent) in the 2012 election[9], 22.9 per cent (30.7 per cent) in the 2016 election, and 41 per cent (the figure is similar if we remove the 2 pp threshold) in the 2020 election. Because we only possess static data about respondents' pre-election beliefs and attitudes, we are not in a position to say if (and which) voters eventually updated their expectations. However, it appears that a large portion of voters in 2000, 2004, 2016, and particularly 2020, were ready to maintain cognitive consistency in the face of disconfirming evidence simply by ignoring the polls (assuming they paid any attention to them).

Exploring the relationship between vote intention polls and voters' expectations without individual-level data on poll exposure requires making a number of assumptions: that (1) individuals have been exposed to polls, (2) they recall polling results correctly, (3) they trust polls and/or find them useful in deciding who will win. Furthermore, and in relation to point 3, the interpretation of polling results is not immune to motivated reasoning. According to Madson and Hillygus (2020, 1068), "the American public evaluates the credibility of a poll based on the extent to which the poll's results offers support to their predispositions" (see also Kuru, Pasek and Traugott 2020). Not only are individuals more likely to believe results favouring their preferred party or candidate, but they also tend to display *stronger* enthusiasm for their candidate after being exposed to *displeasing* information (i.e., a large margin of victory for the opposing candidate). Therefore, it cannot be assumed that polls will be treated as objective pieces of information by voters or that the information they contain will be accurately remembered or properly interpreted. Unfortunately, we do not have any information about respondents' exposure to and confidence in vote intention surveys in the 1996–2020 ANES. In an era of polarized politics (Iyengar et al., 2019)[10] and in light of Madson and Hillygus's (2020) results, it is reasonable to question the influence of polls on voters' expectations. Unless one candidate is clearly ahead of the other(s) for most or all of the campaign (as in 1996), individuals might be increasingly likely to let their preferences drive their expectations. Furthermore, as McGregor (1938, 192) argued in one of the very first studies of voter's expectations, in order to influence one's forecast, a desired outcome must be perceived as realistically possible: in the absence of ambiguity, "[e]ven an intense wish would be inoperative." Since close presidential elections have become the norm over the last two decades (all elections covered by the present study have been won by single-digit popular vote margins), there is now perhaps ample room for ambiguity.

[9] Barack Obama's and Mitt Romney's leads were often quite small—additionally, a great number of ANES respondents took the survey on October 16 when Romney had an average lead of less than 0.8 percentage points over Obama. This is why the figures (1.4 per cent vs 49.8 per cent) are quite different with and without the 2 pp threshold.

[10] Polarization can be seen in respondents' willingness to declare the candidate of the opposing party as the likely winner. In 1996, 35.3 per cent of ANES respondents identifying with the Democratic or Republican Party mentioned the presidential candidate of the opposing party as the most likely winner. About 25.3 and 30.5 per cent of Democratic and Republican respondents did so in 2000 and 2004, respectively. In the three most recent elections, respondents identifying with one of the two major parties were less likely to give the upper hand to the candidate of the opposing party: it was the case of only 15.2 per cent of (Democratic and Republican) respondents in 2012, 22.2 per cent in 2016, and 14.5 per cent in 2020.

Figure H.1. Daily Averages of Nationwide Presidential Vote Intentions for Democratic and Republican Candidates in the 90 Days Preceding the Election, 1996–2020

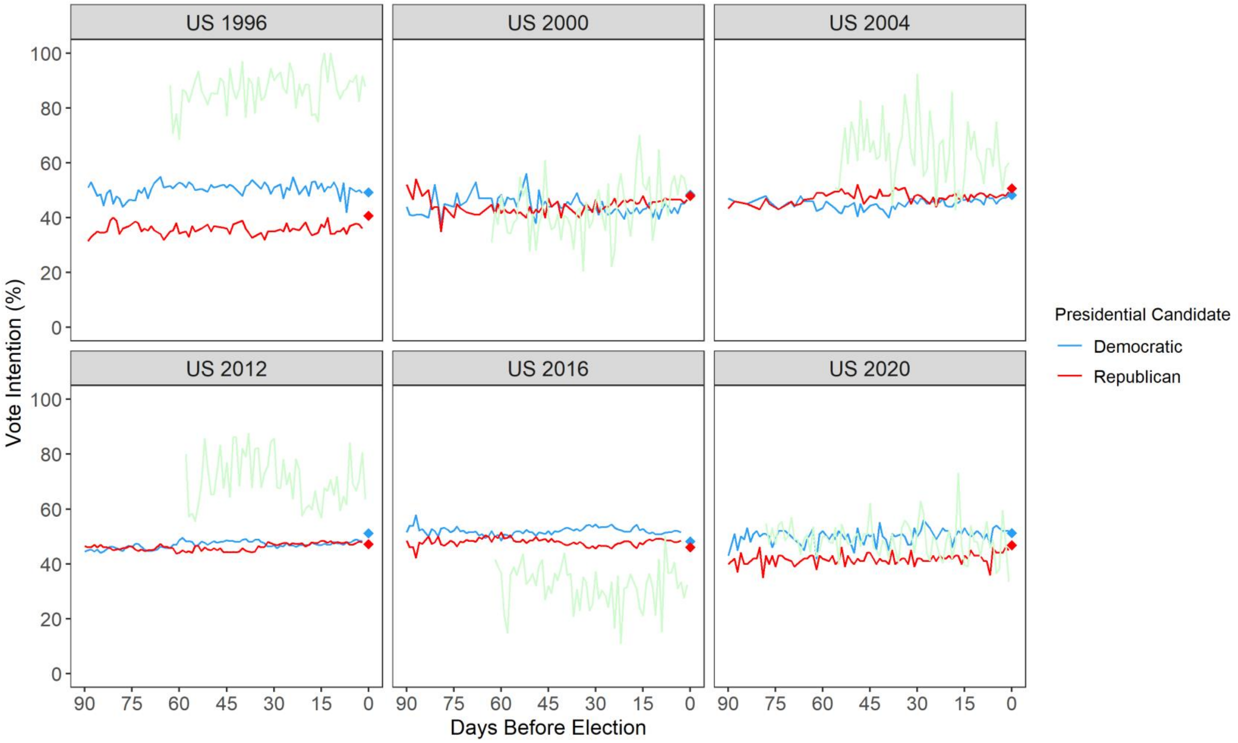

Note. The pale green line shows the percentage of correct forecasts among ANES respondents. Daily poll averages between 1996 and 2016 were taken from Jennings and Wlezien (2017). For the 2020 U.S. presidential election, the necessary data were retrieved from Wikipedia.

Figure H.2. Daily Democratic Nationwide Popular Vote Leads in the 90 Days Preceding the Election, 1996–2020

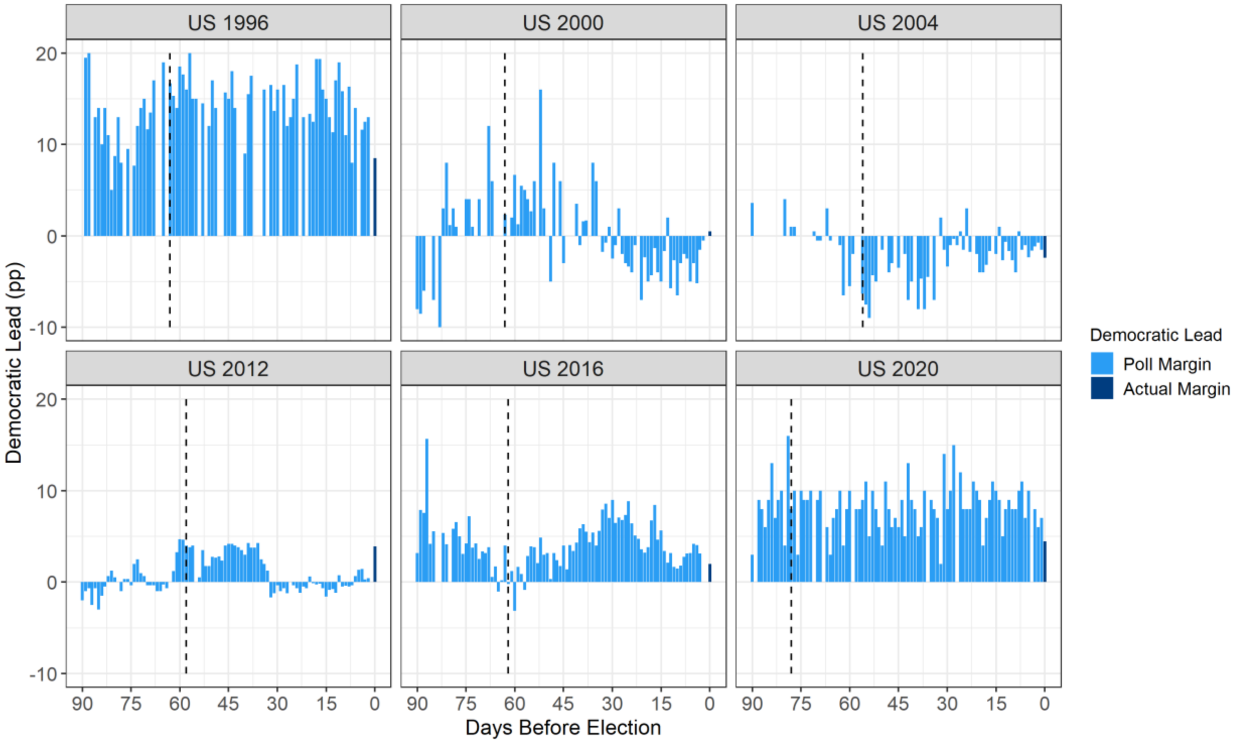

Note. The dashed line indicates the beginning of the ANES pre-election interview period.

I Suspicious Thinking: Additional Details and Analyses

In the 1996, 2000, and 2004 ANES, political cynicism and social mistrust items were measured in the post-election surveys only (with a few exceptions). In 2012, 2016, and 2020, the ANES included both pre- and post-election measures of political cynicism. Therefore, additional analyses were conducted using post-election indicators.

Additional analyses were conducted for the 1996–2004 elections with the inclusion of post-election measures of political cynicism and social mistrust.[11] See tables I.1.1–I.1.3 (party identification) and tables I.2.1–I.2.3 (reported vote). Models 1.1 and 2.1 show the regression outputs with controlling for political cynicism and social mistrust for perceived election fairness and satisfaction with democracy, respectively. Models 1.2 and 2.2 show the same analyses, this time with the inclusion of political cynicism and social mistrust as controls. Generally speaking, political cynicism, but not social mistrust, is a highly significant predictor of both perceived electoral integrity and satisfaction with democracy. Cynical individuals are considerably less likely to harbour positive attitudes or opinions toward election fairness and the way democracy works in the US. For the 2012–2020 elections, the main models (including pre-election measures of cynicism) were compared to models using only post-election measures of cynicism and both pre- *and* post-election measures. See tables I.1.4–I.1.6 (party identification) and tables I.2.4–I.2.6 (reported vote). Overall, these additional analyses do not alter any of the conclusions presented in the paper.

I.1 Party Identification

Table I.1.1. Perceived Electoral Integrity and Satisfaction with Democracy, 1996 U.S. Presidential Election

	FAIR		SWD	
	**Model 1.1** Without	**Model 1.2** With	**Model 2.1** Without	**Model 2.2** With
Electoral status				
Loser (R)				
Independent	-0.04	-0.04	-0.14^{+}	-0.13
Winner	0.23^{***}	0.17^{*}	0.01	-0.01
Unexpected outcome?				
Yes	-0.14	-0.20	0.02	0.01
2-way interaction				
Independent × Unexpected	-0.24	-0.18	0.16	0.21
Winner × Unexpected	0.06	0.16	0.16	0.20
Suspicious thinking				
Political cynicism		-0.98^{***}		-0.71^{***}
Social mistrust		-0.08		-0.06
Economic evaluations				
Egotropic			0.26^{*}	0.23^{+}
Sociotropic			0.60^{***}	0.50^{***}
Information				
Political knowledge	0.43^{**}	0.42^{**}	0.44^{***}	0.49^{***}
Constant	2.50^{***}	3.27^{***}	1.96^{***}	2.51^{***}
Sociodemographics	Yes	Yes	Yes	Yes
Region fixed effects	Yes	Yes	Yes	Yes
Observations	1,363	1,291	1,325	1,264
R^2	0.06	0.10	0.11	0.15

Note. Ordinary Least Squares regressions. Significance levels: + $p < 0.10$; * $p < 0.05$; ** $p < 0.01$; *** $p < 0.001$. FAIR = Perceived electoral integrity. SWD = Satisfaction with democracy. R = Reference category.

[11] Social mistrust was measured in the pre-election wave of the 1996 ANES.

Table I.1.2. Perceived Electoral Integrity and Satisfaction with Democracy, 2000 U.S. Presidential Election

	FAIR		SWD	
	**Model 1.1** Without	**Model 1.2** With	**Model 2.1** Without	**Model 2.2** With
Electoral status				
Loser (R)				
Independent	-0.29	-0.10	-0.54*	-0.43
Winner	0.29	0.42*	0.05	0.08
Unexpected outcome?				
Yes	-0.17	-0.02	0.01	0.10
2-way interaction				
Independent × Unexpected	0.35	0.19	0.28	0.07
Winner × Unexpected	0.30	0.11	-0.01	-0.10
Suspicious thinking				
Political cynicism		-1.41***		-1.20***
Social mistrust		-0.09		-0.09
Economic evaluations				
Egotropic			-0.15	-0.05
Sociotropic			0.76**	0.56+
Information				
Political knowledge	0.22	0.24	0.66**	0.67**
Constant	1.04***	1.85***	2.04***	3.01***
Sociodemographics	Yes	Yes	Yes	Yes
Region fixed effects	Yes	Yes	Yes	Yes
Observations	624	596	294	283
R^2	0.14	0.19	0.18	0.25

Note. Ordinary Least Squares regressions. Significance levels: + $p < 0.10$; * $p < 0.05$; ** $p < 0.01$; *** $p < 0.001$. FAIR = Perceived electoral integrity. SWD = Satisfaction with democracy. R = Reference category.

Table I.1.3. Perceived Electoral Integrity and Satisfaction with Democracy, 2004 U.S. Presidential Election

	FAIR		SWD	
	**Model 1.1** Without	**Model 1.2** With	**Model 2.1** Without	**Model 2.2** With
Electoral status				
Loser (R)				
Independent	-0.03	-0.04	-0.01	0.01
Winner	0.62***	0.54***	0.25***	0.26***
Unexpected outcome?				
Yes	-0.42***	-0.41***	-0.23**	-0.23**
2-way interaction				
Independent × Unexpected	-0.04	-0.01	0.17	0.13
Winner × Unexpected	-0.11	-0.11	0.14	0.12
Suspicious thinking				
Political cynicism		-1.04***		-0.76***
Social mistrust		0.01		-0.10
Economic evaluations				
Egotropic			0.21	0.14
Sociotropic			0.76***	0.59***
Information				
Political knowledge	0.02	0.09	0.16	0.17
Constant	2.25***	2.90***	2.10***	2.80***
Sociodemographics	Yes	Yes	Yes	Yes
Region fixed effects	Yes	Yes	Yes	Yes
Observations	926	879	861	826
R^2	0.23	0.26	0.21	0.24

Note. Ordinary Least Squares regressions. Significance levels: + $p < 0.10$; * $p < 0.05$; ** $p < 0.01$; *** $p < 0.001$. FAIR = Perceived electoral integrity. SWD = Satisfaction with democracy. R = Reference category.

Table I.1.4. Perceived Electoral Integrity and Satisfaction with Democracy, 2012 U.S. Presidential Election

	FAIR			SWD		
	**M1.1** Pre	**M1.2** Post	**M1.3** Pre/Post	**M2.1** Pre	**M2.2** Post	**M2.3** Pre/Post
Electoral status						
Loser (R)						
Independent	-0.04	0.08	0.07	-0.06	-0.13	-0.13
Winner	0.00	0.08	0.06	0.02	-0.06	-0.07
Unexpected outcome?						
Yes	-0.13**	-0.12	-0.12+	0.02	-0.02	-0.03
2-way interaction						
Independent × Unexpected	0.06	-0.05	-0.05	-0.18+	-0.14	-0.15
Winner × Unexpected	-0.02	-0.10	-0.09	-0.08	0.02	0.02
Suspicious thinking						
Conspiracy beliefs	-1.07***	-1.11***	-1.09***	-0.46***	-0.55***	-0.52***
Political cynicism (pre)	-0.70***			-1.13***		
Political cynicism (post)		-0.36**			-0.70***	
Political cynicism (pre/post)			-0.59***			-1.07***
Social mistrust	-0.05**	-0.07**	-0.06*	-0.04*	-0.03	-0.02
Economic evaluations						
Egotropic				0.17*	0.11	0.15
Sociotropic				0.35**	0.56***	0.44**
Information						
Political knowledge	-0.02	-0.04	-0.04	-0.11+	-0.15+	-0.14+
Constant	3.54***	3.34***	3.38***	3.43***	3.22***	3.29***
Sociodemographics	Yes	Yes	Yes	Yes	Yes	Yes
Region fixed effects	Yes	Yes	Yes	Yes	Yes	Yes
Observations	4,424	2,260	2,241	4,314	2,205	2,186
R^2	0.19	0.20	0.20	0.17	0.15	0.16

Note. Ordinary Least Squares regressions. Significance levels: + $p < 0.10$; * $p < 0.05$; ** $p < 0.01$; *** $p < 0.001$. FAIR = Perceived electoral integrity. SWD = Satisfaction with democracy. R = Reference category.

Table I.1.5. Perceived Electoral Integrity and Satisfaction with Democracy, 2016 U.S. Presidential Election

	FAIR			SWD		
	**M1.1** Pre	**M1.2** Post	**M1.3** Pre/Post	**M2.1** Pre	**M2.2** Post	**M2.3** Pre/Post
Electoral status						
Loser (R)						
Independent	0.04	0.00	0.03	-0.08	-0.07	-0.07
Winner	0.04	0.00	0.01	0.19*	0.15+	0.15+
Unexpected outcome?						
Yes	-0.10	-0.07	-0.08	-0.11	-0.09	-0.10
2-way interaction						
Independent × Unexpected	-0.17	-0.14	-0.16	0.05	0.06	0.04
Winner × Unexpected	0.10	0.08	0.09	0.09	0.09	0.10
Suspicious thinking						
Conspiracy beliefs	-0.18**	-0.21**	-0.17*	0.05	0.08	0.11
Political cynicism (pre)	-0.59***			-0.63***		
Political cynicism (post)		-0.43***			-0.88***	
Political cynicism (pre/post)			-0.63***			-1.02***
Social mistrust	-0.08***	-0.09***	-0.08***	-0.02	-0.02	-0.02
Economic evaluations						
Egotropic				-0.10	-0.05	-0.10
Sociotropic				0.52***	0.65***	0.55***
Information						
Political knowledge	0.23**	0.20**	0.23**	0.11	0.10	0.13+
Constant	3.14***	3.10***	3.14***	2.66***	2.74***	2.83***
Sociodemographics	Yes	Yes	Yes	Yes	Yes	Yes
Region fixed effects	Yes	Yes	Yes	Yes	Yes	Yes
Observations	3,098	3,102	3,081	3,042	3,049	3,029
R^2	0.14	0.12	0.13	0.11	0.12	0.13

Note. Ordinary Least Squares regressions. Significance levels: + $p < 0.10$; * $p < 0.05$; ** $p < 0.01$; *** $p < 0.001$. FAIR = Perceived electoral integrity. SWD = Satisfaction with democracy. R = Reference category.

Table I.1.6. Perceived Electoral Integrity and Satisfaction with Democracy, 2020 U.S. Presidential Election

	FAIR			SWD		
	**M1.1** Pre	**M1.2** Post	**M1.3** Pre/Post	**M2.1** Pre	**M2.2** Post	**M2.3** Pre/Post
Electoral status						
Loser (R)						
Independent	0.07	0.07	0.07	-0.17*	-0.16^{+}	-0.17*
Winner	0.26***	0.27***	0.26***	-0.09	-0.09	-0.09
Unexpected outcome?						
Yes	-0.51***	-0.51***	-0.51***	-0.08	-0.07	-0.07
2-way interaction						
Independent × Unexpected	0.18^{+}	0.17^{+}	0.17^{+}	0.01	0.00	0.02
Winner × Unexpected	0.21**	0.20**	0.21**	-0.00	-0.02	-0.01
Suspicious thinking						
Conspiracy beliefs	-0.58***	-0.61***	-0.59***	-0.45***	-0.37***	-0.32***
Political cynicism (pre)	-0.07			-0.49***		
Political cynicism (post)		0.07			-0.78***	
Political cynicism (pre/post)			0.02			-0.97***
Social mistrust	-0.06***	-0.07***	-0.07***	-0.01	-0.01	-0.01
Economic evaluations						
Egotropic				0.06	0.06	0.06
Sociotropic				0.29***	0.33***	0.30***
Information						
Political knowledge	0.36***	0.35***	0.35***	-0.14^{+}	-0.14*	-0.14^{+}
Constant	3.10***	3.03***	3.05***	2.96***	3.10***	3.18***
Sociodemographics	Yes	Yes	Yes	Yes	Yes	Yes
Region fixed effects	Yes	Yes	Yes	Yes	Yes	Yes
Observations	6,543	6,552	6,499	6,474	6,494	6,451
R^2	0.30	0.30	0.30	0.08	0.09	0.09

Note. Ordinary Least Squares regressions. Significance levels: + $p < 0.10$; * $p < 0.05$; ** $p < 0.01$; *** $p < 0.001$. FAIR = Perceived electoral integrity. SWD = Satisfaction with democracy. R = Reference category.

I.2 Reported Vote

Table I.2.1. Perceived Electoral Integrity and Satisfaction with Democracy, 1996 U.S. Presidential Election

	FAIR		SWD	
	**Model 1.1** Without	**Model 1.2** With	**Model 2.1** Without	**Model 2.2** With
Electoral status				
Loser (R)				
Loser (R)	0.00	0.00	0.00	0.00
Non-voter	-0.11	-0.13	-0.15*	-0.15*
Winner	0.34***	0.24***	0.05	0.00
Unexpected outcome?				
Yes	-0.08	-0.15	0.00	-0.01
2-way interaction				
Non-voter × Unexpected	-0.09	-0.11	0.19	0.18
Winner × Unexpected	0.04	0.36	0.09	0.21
Suspicious thinking				
Political cynicism		-0.96***		-0.71***
Social mistrust		-0.06		-0.05
Economic evaluations				
Egotropic			0.25*	0.23+
Sociotropic			0.58***	0.49***
Information				
Political knowledge	0.31*	0.32*	0.39***	0.45***
Constant	2.66***	3.40***	2.06***	2.61***
Sociodemographics	Yes	Yes	Yes	Yes
Region fixed effects	Yes	Yes	Yes	Yes
Observations	1,363	1,291	1,325	1,264
R^2	0.08	0.12	0.12	0.16

Note. Ordinary Least Squares regressions. Significance levels: + $p < 0.10$; * $p < 0.05$; ** $p < 0.01$; *** $p < 0.001$. FAIR = Perceived electoral integrity. SWD = Satisfaction with democracy. R = Reference category.

Table I.2.2. Perceived Electoral Integrity and Satisfaction with Democracy, 2000 U.S. Presidential Election

	FAIR		SWD	
	**Model 1.1** Without	**Model 1.2** With	**Model 2.1** Without	**Model 2.2** With
Electoral status				
Loser (R)				
Non-voter	-0.45^{*}	-0.36^{+}	-0.12	-0.15
Winner	0.30	0.33^{+}	0.12	0.18
Unexpected outcome?				
Yes	-0.23	-0.18	0.04	0.12
2-way interaction				
Non-voter × Unexpected	0.40	0.40	0.06	0.03
Winner × Unexpected	0.43	0.43	-0.03	-0.07
Suspicious thinking				
Political cynicism		-1.53^{***}		-1.30^{***}
Social mistrust		-0.09		-0.07
Economic evaluations				
Egotropic			-0.20	-0.04
Sociotropic			0.82^{**}	0.55^{+}
Information				
Political knowledge	0.06	0.14	0.59^{*}	0.55^{*}
Constant	1.26^{***}	2.21^{***}	1.98^{***}	3.12^{***}
Sociodemographics	Yes	Yes	Yes	Yes
Region fixed effects	Yes	Yes	Yes	Yes
Observations	621	592	295	283
R^2	0.16	0.22	0.15	0.23

Note. Ordinary Least Squares regressions. Significance levels: + $p < 0.10$; * $p < 0.05$; ** $p < 0.01$; *** $p < 0.001$. FAIR = Perceived electoral integrity. SWD = Satisfaction with democracy. R = Reference category.

Table I.2.3. Perceived Electoral Integrity and Satisfaction with Democracy, 2004 U.S. Presidential Election

	FAIR		SWD	
	**Model 1.1** Without	**Model 1.2** With	**Model 2.1** Without	**Model 2.2** With
Electoral status				
Loser (R)				
Non-voter	0.42**	0.31*	0.16+	0.15
Winner	0.79***	0.65***	0.31***	0.30***
Unexpected outcome?				
Yes	-0.27*	-0.30*	-0.26**	-0.28**
2-way interaction				
Non-voter × Unexpected	-0.22	-0.15	0.31*	0.29*
Winner × Unexpected	0.08	0.05	0.44**	0.40*
Suspicious thinking				
Political cynicism		-0.93***		-0.64***
Social mistrust		-0.01		-0.13*
Economic evaluations				
Egotropic			0.19	0.14
Sociotropic			0.70***	0.56***
Information				
Political knowledge	0.15	0.17	0.26*	0.25*
Constant	2.07***	2.73***	2.00***	2.66***
Sociodemographics	Yes	Yes	Yes	Yes
Region fixed effects	Yes	Yes	Yes	Yes
Observations	923	877	856	822
R^2	0.24	0.26	0.23	0.26

Note. Ordinary Least Squares regressions. Significance levels: + $p < 0.10$; * $p < 0.05$; ** $p < 0.01$; *** $p < 0.001$. FAIR = Perceived electoral integrity. SWD = Satisfaction with democracy. R = Reference category.

Table I.2.4. Perceived Electoral Integrity and Satisfaction with Democracy, 2012 U.S. Presidential Election

	FAIR			SWD		
	**M1.1** Pre	**M1.2** Post	**M1.3** Pre/Post	**M2.1** Pre	**M2.2** Post	**M2.3** Pre/Post
Electoral status						
Loser (R)						
Non-voter	-0.14^{+}	0.04	0.02	0.31***	0.35***	0.33***
Winner	0.02	0.01	-0.01	0.22***	0.20*	0.18*
Unexpected outcome?						
Yes	-0.15**	-0.16*	-0.17*	0.20***	0.23**	0.21*
2-way interaction						
Non-voter × Unexpected	0.11	-0.11	-0.09	-0.45***	-0.55***	-0.52***
Winner × Unexpected	0.08	0.08	0.10	-0.24*	-0.16	-0.14
Suspicious thinking						
Conspiracy beliefs	-1.00***	-1.09***	-1.06***	-0.45***	-0.54***	-0.51***
Political cynicism (pre)	-0.71***			-1.09***		
Political cynicism (post)		-0.31*			-0.69***	
Political cynicism (pre/post)			-0.56***			-1.09***
Social mistrust	-0.05**	-0.07**	-0.07**	-0.05*	-0.03	-0.02
Economic evaluations						
Egotropic				0.21*	0.14	0.17
Sociotropic				0.25*	0.44**	0.33*
Information						
Political knowledge	-0.03	-0.03	-0.02	-0.11^{+}	-0.14	-0.12
Constant	3.61***	3.41***	3.47***	3.24***	2.97***	3.08***
Sociodemographics	Yes	Yes	Yes	Yes	Yes	Yes
Region fixed effects	Yes	Yes	Yes	Yes	Yes	Yes
Observations	4,090	2,078	2,058	3,992	2,026	2,008
R^2	0.19	0.19	0.19	0.17	0.15	0.16

Note. Ordinary Least Squares regressions. Significance levels: + $p < 0.10$; * $p < 0.05$; ** $p < 0.01$; *** $p < 0.001$. FAIR = Perceived electoral integrity. SWD = Satisfaction with democracy. R = Reference category.

Table I.2.5. Perceived Electoral Integrity and Satisfaction with Democracy, 2016 U.S. Presidential Election

	FAIR			SWD		
	**M1.1** Pre	**M1.2** Post	**M1.3** Pre/Post	**M2.1** Pre	**M2.2** Post	**M2.3** Pre/Post
Electoral status						
Loser (R)						
Non-voter	0.10	0.09	0.08	0.06	0.05	0.03
Winner	0.08	0.06	0.05	0.25***	0.21**	0.21**
Unexpected outcome?						
Yes	-0.02	0.02	-0.01	-0.09	-0.07	-0.10
2-way interaction						
Non-voter × Unexpected	-0.28**	-0.29**	-0.28**	-0.01	0.00	0.01
Winner × Unexpected	0.06	0.02	0.05	0.09	0.08	0.11
Suspicious thinking						
Conspiracy beliefs	-0.19**	-0.21**	-0.17*	-0.00	0.04	0.06
Political cynicism (pre)	-0.60***			-0.65***		
Political cynicism (post)		-0.44***			-0.91***	
Political cynicism (pre/post)			-0.64***			-1.05***
Social mistrust	-0.08***	-0.08***	-0.08***	-0.02	-0.02	-0.02
Economic evaluations						
Egotropic				-0.07	-0.03	-0.07
Sociotropic				0.55***	0.68***	0.58***
Information						
Political knowledge	0.21**	0.18**	0.21**	0.09	0.09	0.12
Constant	3.14***	3.07***	3.14***	2.62***	2.70***	2.80***
Sociodemographics	Yes	Yes	Yes	Yes	Yes	Yes
Region fixed effects	Yes	Yes	Yes	Yes	Yes	Yes
Observations	3,096	3,100	3,079	3,038	3,045	3,025
R^2	0.14	0.13	0.14	0.10	0.12	0.13

Note. Ordinary Least Squares regressions. Significance levels: + $p < 0.10$; * $p < 0.05$; ** $p < 0.01$; *** $p < 0.001$. FAIR = Perceived electoral integrity. SWD = Satisfaction with democracy. R = Reference category.

Table I.2.6. Perceived Electoral Integrity and Satisfaction with Democracy, 2020 U.S. Presidential Election

	FAIR			SWD		
	**M1.1** Pre	**M1.2** Post	**M1.3** Pre/Post	**M2.1** Pre	**M2.2** Post	**M2.3** Pre/Post
Electoral status						
Loser (R)						
Non-voter	0.33^{***}	0.33^{***}	0.33^{***}	0.07	0.11	0.09
Winner	0.46^{***}	0.47^{***}	0.47^{***}	-0.03	-0.01	-0.03
Unexpected outcome?						
Yes	-0.34^{***}	-0.33^{***}	-0.34^{***}	0.00	0.03	0.01
2-way interaction						
Non-voter × Unexpected	-0.24^{*}	-0.24^{**}	-0.24^{*}	-0.17^{*}	-0.22^{*}	-0.19^{*}
Winner × Unexpected	0.24^{**}	0.22^{**}	0.23^{**}	-0.01	-0.04	-0.01
Suspicious thinking						
Conspiracy beliefs	-0.55^{***}	-0.56^{***}	-0.55^{***}	-0.46^{***}	-0.37^{***}	-0.32^{***}
Political cynicism (pre)	-0.08			-0.50^{***}		
Political cynicism (post)		0.04			-0.80^{***}	
Political cynicism (pre/post)			-0.00			-0.99^{***}
Social mistrust	-0.06^{**}	-0.06^{***}	-0.06^{***}	-0.01	-0.01	-0.01
Economic evaluations						
Egotropic				0.07	0.06	0.07
Sociotropic				0.32^{***}	0.36^{***}	0.33^{***}
Information						
Political knowledge	0.32^{***}	0.30^{***}	0.31^{***}	-0.12	-0.11	-0.11
Constant	2.93^{***}	2.87^{***}	2.89^{***}	2.83^{***}	2.96^{***}	3.06^{***}
Sociodemographics	Yes	Yes	Yes	Yes	Yes	Yes
Region fixed effects	Yes	Yes	Yes	Yes	Yes	Yes
Observations	6,510	6,519	6,467	6,443	6,462	6,420
R^2	0.32	0.32	0.32	0.08	0.09	0.09

Note. Ordinary Least Squares regressions. Significance levels: + $p < 0.10$; * $p < 0.05$; ** $p < 0.01$; *** $p < 0.001$. FAIR = Perceived electoral integrity. SWD = Satisfaction with democracy. R = Reference category.

References

American National Election Study. 1996. "1996 Time Series Study." American National Election Study (ANES). https://electionstudies.org/data-center/1996-time-series-study/.

American National Election Study. 2000. "2000 Time Series Study." American National Election Study (ANES). https://electionstudies.org/data-center/2000-time-series-study/.

American National Election Study. 2004. "2004 Time Series Study." American National Election Study (ANES). https://electionstudies.org/data-center/2004-time-series-study/.

American National Election Study. 2012. "2012 Time Series Study." American National Election Study (ANES). https://electionstudies.org/data-center/2012-time-series-study/.

American National Election Study. 2016. "2016 Time Series Study." American National Election Study (ANES). https://electionstudies.org/data-center/2016-time-series-study/.

American National Election Study. 2020. "2020 Time Series Study." American National Election Study (ANES). https://electionstudies.org/data-center/2020-time-series-study/.

Ansolabehere, Stephen and Shanto Iyengar. 1994. "Of Horseshoes and Horse Races: Experimental Studies of the Impact of Poll Results on Electoral Behavior." *Political Communication* 11(4):413–430.

Blais, André, Elisabeth Gidengil and Anja Kilibarda. 2017. "Partisanship, Information, and Perceptions of Government Corruption." *International Journal of Public Opinion Research* 29(1):95–110.

Blais, André and François Gélineau. 2007. "Winning, Losing and Satisfaction with Democracy." *Political Studies* 55(2):425–441.

Booth, Eric. 2003. "NEARMRG: Stata Module to Provide Nearest-Match Merging of Datasets." Statistical Software Components S434901, Boston College Department of Economics, revised 7 Feb 2012. https://ideas.repec.org/c/boc/bocode/s434901.html.

Brants, Kees. 2013. Trust, Cynicism, and Responsiveness: The Uneasy Situation of Journalism in Democracy. In *Rethinking Journalism: Trust and Participation in a Transformed Media Landscape*, ed. Chris Peters and M. J. Broersma. Abingdon: Routledge, pp. 15–27.

Craig, Stephen C., Michael D. Martinez, Jason Gainous and James G. Kane. 2006. "Winners, Losers, and Election Context: Voter Responses to the 2000 Presidential Election." *Political Research Quarterly* 59(4):579–592.

Daschmann, Gregor. 2000. "Vox Pop and Polls: The Impact of Poll Results and Voter Statements in the Media on the Perception of a Climate of Opinion." *International Journal of Public Opinion Research* 12(2):160–181.

Edelson, Jack, Alexander Alduncin, Christopher Krewson, James A. Sieja and Joseph E. Uscinski. 2017. "The Effect of Conspiratorial Thinking and Motivated Reasoning on Belief in Election Fraud." *Political Research Quarterly* 70(4):933–946.

Enders, Adam M. and Steven M. Smallpage. 2019. "Informational Cues, Partisan-Motivated Reasoning, and the Manipulation of Conspiracy Beliefs." *Political Communication* 36(1):83–102.

Faas, Thorsten, Christian Mackenrodt and Rüdiger Schmitt-Beck. 2008. "Polls that Mattered: Effects of Media Polls on Voters' Coalition Expectations and Party Preferences in the 2005 German Parliamentary Election." *International Journal of Public Opinion Research* 20(3):299–325.

Fahey, James J., Trevor J. Allen and Hannah M. Alarian. 2022. "When Populists Win: How Right-Wing Populism Affects Democratic Satisfaction in the U.K. and Germany." *Electoral Studies* 77:102469.

Ferić, Ivana and Vesna Lamza Posavec. 2013. "Opinion Polls, Voters' Intentions and Expectations in the 2011 Croatian Parliamentary Elections." *European Quarterly of Political Attitudes and Mentalities* 2(4):4–15.

Halliez, Adrien A. and Judd R. Thornton. 2022. “The Winner-Loser Satisfaction Gap in the Absence of a Clear Outcome.” *Party Politics* (forthcoming):1–10.

Irwin, Galen A. and Joop J. M. van Holsteyn. 2002. “According to the Polls: The Influence of Opinion Polls on Expectations.” *Public Opinion Quarterly* 66(1):92–104.

Iyengar, Shanto, Yphtach Lelkes, Matthew Levendusky, Neil Malhotra and Sean J. Westwood. 2019. “The Origins and Consequences of Affective Polarization in the United States.” *Annual Review of Political Science* 22:129–146.

Jennings, Will and Christopher Wlezien. 2017. “Replication Data for: Election Polling Errors Across Time and Space.” Harvard Dataverse. Datafile Version 3. https://doi.org/10.7910/DVN/8421DX.

Kaid, Lynda Lee, Mitchell McKinney and John C. Tedesco. 2000. *Civic Dialogue in the 1996 Presidential Campaign Candidate, Media, and Public Voices*. New York: Hampton Press.

Keith, Bruce E., David B. Magleby, Candice J. Nelson, Elizabeth Orr, Mark C. Westlye and Raymond E. Wolfinger. 1992. *The Myth of the Independent Voter*. Berkeley: University of California Press.

Kornberg, Allan and Harold D. Clarke. 1992. *Citizens and Community: Political Support in a Representative Democracy*. Cambridge: Cambridge University Press.

Kuru, Ozan, Josh Pasek and Michael W. Traugott. 2020. “When Polls Disagree: How Competitive Results and Methodological Quality Shape Partisan Perceptions of Polls and Electoral Predictions.” *International Journal of Public Opinion Research* 32(3):586–603.

Lamberty, Pia K., Jens H. Hellmann and Aileen Oeberst. 2018. “The Winner Knew It All? Conspiracy Beliefs and Hindsight Perspective After the 2016 US General Election.” *Personality and Individual Differences* 123:236–240.

Madson, Gabriel J. and D. Sunshine Hillygus. 2020. “All the Best Polls Agree with Me: Bias in Evaluations of Political Polling.” *Political Behavior.* 42:1055–1072.

Magleby, David B., Candice J. Nelson and Mark C. Westlye. 2011. The Myth of the Independent Voter Revisited. In *Facing the Challenge of Democracy: Explorations in the Analysis of Public Opinion and Political Participation*, ed. Paul M. Sniderman and Benjamin Highton. Princeton: Princeton University Press, pp. 238–264.

McGregor, Douglas. 1938. “The Major Determinants of the Prediction of Social Events.” *Journal of Abnormal and Social Psychology* 33(2):179–204.

Meffert, Michael F., Sascha Huber, Thomas Gschwend and Franz Urban Pappi. 2011. “More than Wishful Thinking: Causes and Consequences of Voters’ Electoral Expectations About Parties and Coalitions.” *Electoral Studies* 30(4):804–815.

Miller, Arthur H. 1974. “Political Issues and Trust in Government: 1964–1970.” *American Political Science Review* 68(3):951–972.

Miller, Joanne M., Kyle L. Saunders and Christina E. Farhart. 2016. “Conspiracy Endorsement as Motivated Reasoning: The Moderating Roles of Political Knowledge and Trust.” *American Journal of Political Science* 60(4):824–844.

Morwitz, Vicki G. and Carol Pluzinski. 1996. “Do Polls Reflect Opinions or Do Opinions Reflect Polls? The Impact of Political Polling on Voters’ Expectations, Preferences, and Behavior.” *Journal of Consumer Research* 23(1):53–67.

Rijkhoff, Sanne A. M. 2018. “Still Questioning Cynicism.” *Society* 55(4):333–340.

Rudolph, Thomas. 2021. “Populist Anger, Donald Trump, and the 2016 Election.” *Journal of Elections, Public Opinion and Parties* 31(1):33–58.

Schneiker, Andrea. 2020. “Populist Leadership: The Superhero Donald Trump as Savior in Times of Crisis.” *Political Studies* 68(4):857–874.

Uscinski, Joseph E., Casey Klofstad and Matthew D. Atkinson. 2016. “What Drives Conspiratorial Beliefs? The Role of Informational Cues and Predispositions.” *Political Research Quarterly* 69(1):57–71.
